# A novel *FLNC* frameshift and an *OBSCN* variant in a family with distal muscular dystrophy

**DOI:** 10.1371/journal.pone.0186642

**Published:** 2017-10-26

**Authors:** Daniela Rossi, Johanna Palmio, Anni Evilä, Lucia Galli, Virginia Barone, Tracy A. Caldwell, Rachel A. Policke, Esraa Aldkheil, Christopher E. Berndsen, Nathan T. Wright, Edoardo Malfatti, Guy Brochier, Enrico Pierantozzi, Albena Jordanova, Velina Guergueltcheva, Norma Beatriz Romero, Peter Hackman, Bruno Eymard, Bjarne Udd, Vincenzo Sorrentino

**Affiliations:** 1 Molecular Medicine Section, Department of Molecular and Developmental Medicine, University of Siena and Azienda Ospedaliera Universitaria Senese, Siena, Italy; 2 Neuromuscular Research Center, Tampere University and University Hospital, Tampere, Finland; 3 Folkhälsan Institute of Genetics and Department of Medical Genetics, Haartman Institute, University of Helsinki, Helsinki, Finland; 4 Department of Chemistry and Biochemistry, James Madison University, Harrisonburg, Virginia, United States of America; 5 Neuromuscular Morphology Unit, and Reference Center for Neuromuscular Diseases, Myology Institute, Groupe Hospitalier La Pitié-Salpêtrière, Paris, France; 6 Molecular Neurogenomics Group, University of Antwerp, Antwerp, Belgium; 7 Molecular Medicine Center, Department of Medical Chemistry and Biochemistry, Medical University-Sofia, Sofia, Bulgaria; 8 Department of Neurology, Medical university of Sofia, Sofia, Bulgaria; 9 Department of Neurology, Vaasa Central Hospital, Vaasa, Finland; University of Liverpool, UNITED KINGDOM

## Abstract

A novel *FLNC* c.5161delG (p.Gly1722ValfsTer61) mutation was identified in two members of a French family affected by distal myopathy and in one healthy relative. This *FLNC* c.5161delG mutation is one nucleotide away from a previously reported *FLNC* mutation (c.5160delC) that was identified in patients and in asymptomatic carriers of three Bulgarian families with distal muscular dystrophy, indicating a low penetrance of the *FLNC* frameshift mutations. Given these similarities, we believe that the two *FLNC* mutations alone can be causative of distal myopathy without full penetrance. Moreover, comparative analysis of the clinical manifestations indicates that patients of the French family show an earlier onset and a complete segregation of the disease. As a possible explanation of this, the two French patients also carry a *OBSCN* c.13330C>T (p.Arg4444Trp) mutation. The p.Arg4444Trp variant is localized within the OBSCN Ig59 domain that, together with Ig58, binds to the ZIg9/ZIg10 domains of titin at Z-disks. Structural and functional studies indicate that this OBSCN p.Arg4444Trp mutation decreases titin binding by ~15-fold. On this line, we suggest that the combination of the *OBSCN* p.Arg4444Trp variant and of the *FLNC* c.5161delG mutation, can cooperatively affect myofibril stability and increase the penetrance of muscular dystrophy in the French family.

## Introduction

Distal muscular dystrophies are a group of inherited primary muscle disorders showing progressive muscle atrophy and weakness preferentially in the hands and/or feet [1–4]. They can be classified according to i) clinical features, i.e. early or late adult onset, onset in the hands or feet; ii) magnetic resonance imaging (MRI)-based evidence of an involvement of the anterior or posterior compartment of legs; iii) inheritance pattern; and iv) histopathological findings [4–5]. In recent years, the genes responsible for many distal muscular dystrophies have been identified (S1 Table). Interestingly, in contrast with proximal muscular dystrophies where the mutated genes often encode sarcolemmal proteins, many of the genes identified in distal muscular dystrophies encode proteins associated with the contractile apparatus and Z-disk [4, 6].

Mutations in filamin C (*FLNC*), a gene that encodes a protein associated to the Z-disk, have been reported to cause myopathies inherited as a dominant trait that may present with different clinical and histological features [7–9]. Patients with mutations in the central or C-terminal regions have progressive proximal muscle weakness associated with the presence of aggregates containing desmin, myotilin and other proteins within myofibers, thus resembling typical profiles of myofibrillar myopathy [9], whereas mutations in the N-terminal actin-binding domain cause a dominant distal myopathy without myofibrillar pathology [7]. Interestingly, another non-myofibrillar myopathy with distal muscle involvement has been reported in three related families from Bulgaria carrying a frameshift deletion in *FLNC* (c.5160delC). This mutation was detected in all patients of these families, but also in some healthy relatives [8]. This deletion appears to result in a loss-of-function mechanism leading to haploinsufficiency with absence of protein aggregates. This phenotype differs from other central or C-terminal *FLNC* mutations, which consistently display aggregate formation [9].

Both dominant and recessive mutations in the titin gene *(TTN)* have been associated with human distal dystrophies [10]. Titin is the largest protein in nature extending from the Z-disk to the M-band, and interacts with a number of sarcomeric and signalling proteins [11]. One of these targets is another giant myofibril-associated protein, termed obscurin (OBSCN), that interacts with titin at both the M-band and the Z-disk [12–15]. Mutations in the C-terminal M10 domain of titin, which binds the Ig1 of obscurin, cause Tibial Muscular Dystrophy (TMD) or Udd myopathy in the heterozygous state, and Limb Girdle Muscular Distrophy 2J in the homozygous state. These mutations ablate the interaction between titin and obscurin at the sarcomeric M-band [13–16]. Obscurin and titin also interact at the Z-disk, through Ig58/Ig59 of obscurin and the titin ZIg9/ZIg10. Mutations in these and other regions of *OBSCN* have been associated with different forms of cardiomyopathy [17–18]. Additionally, *Obscn* knockout mice show sarcolemmal fragility and reduced ability to complete intense running sessions [13,19]. Following heavy exercise, the diaphragm muscle of *Obscn* knockout mice presented inflammation and hyper-contractures with evidence of wavy and less defined M-band and H-zone [20]. Despite these ties to muscle organization and maintenance, no mutations in *OBSCN* have been reported in human skeletal muscle diseases.

To verify the hypothesis that *OBSCN* could be involved in skeletal muscle diseases, we performed genetic analysis studies and identified an *OBSCN* c.13330C>T variant resulting in the p.Arg4444Trp substitution in Ig59. This variant was present in the two affected members of a French family and not in any of the available healthy relatives. Whole exome sequencing (WES) of this family also identified a single base deletion in the *FLNC* gene (c.5161delG) causing a frameshift mutation (p.Gly1722ValfsTer61). The *FLNC* mutation was present in the two affected members of the family but also in one healthy relative. These findings resemble the results previously reported by Guergueltcheva and collaborators [8], where the c.5160delC frameshift deletion in *FLNC* was observed in individuals with muscular dystrophy but also in healthy individuals. WES analysis performed on the DNAs from three patients, one healthy member from the Bulgarian families in which the *FLNC* c.5160delC was described and five unrelated normal controls did not reveal any additional gene variant shared by affected individuals. Based on the analogy with previously reported findings [8], we think that the *FLNC* c.5161delG is the causative mutation in the French family reported here. Nevertheless, although the small size of the French family precludes us from reaching a firm conclusion about the pathogenic relevance of the OBSCN p.Arg4444Trp variant, several lines of evidence suggest that it also might play a role in increasing the penetrance and severity of the myopathic phenotype.

## Materials and methods

### Patient data

Mutation screening was initially performed in families where the affected members presented with a clear distal myopathy phenotype transmitted as dominant trait. The study was then extended to a cohort of 110 unrelated patients consisting of 11 cases with predominantly proximal weakness, respiratory failure and/or cardiomyopathy, 8 cases with predominantly proximal myopathy, 26 cases with initial distal muscle involvement subsequently progressing to proximal limb weakness and 65 patients with a clinical history of uncharacterized muscular dystrophy/myopathy. In the French family reported here, the proband (III:3), a descendant of non-consanguineous parents, and her second son (IV:2), had progressive distal lower limb weakness starting at ages 30 and 14, respectively. The proband’s father (II:2) had very late-onset walking difficulties, became wheelchair bound at age 75 and subsequently died from respiratory insufficiency and pneumonia. The paternal grandfather (I:1) became wheelchair-bound at an advanced age. Other family members had no neuromuscular history. The proband and her affected son were followed in Neuromuscular Unit of Salpêtrière Hospital (Paris, France). Among the four children of the proband, one daughter (IV:3) and one son (IV:4), seen at the Neuromuscular Unit at age of 33 and 32, respectively, had a normal clinical examination, with no muscle alteration on MRI. Ethics committee approval and written informed consent were obtained for all patients. This study complies with the ethical standards stated in the 1964 Declaration of Helsinki and was approved by the Coordinating Ethics committee at The Hospital District of Helsinki and Uusimaa.

### Morphological studies of patients’ muscle biopsies

Muscle specimens were cryofixed in OCT compound (Tissue-Tek-Sakura) by immersion in liquid nitrogen-cooled isopentane and stored at −80°C. For conventional histochemical techniques, serial 10 μm-thick transversal sections were stained with haematoxylin and eosin (H&E), reduced nicotinamide adenine dinucleotide (NADH), or decorated with antibodies against desmin, myotilin or alpha B crystallin, as previously described [21]. Electron microscopy studies were performed as previously described [21,22].

### Mutation screening and genotyping

Mutation screening by conventional Sanger sequencing on specific regions of *OBSCN* was performed in all family members for whom DNA was available. In detail, primers were designed using Primer3 software (http://frodo.wi.mit.edu/primer3) to amplify *OBSCN* exon 2 (coding for Ig1) and exons 50, 51 and 52 (coding for Ig58/Ig59), according to sequence NM_052843. Genomic DNA was extracted from peripheral blood leucocytes by standard procedures [22]. Amplified DNA fragments were directly sequenced using an ABI Prism 310 apparatus (Applied Biosystem). Alternatively, Sanger sequencing was performed using DreamTaq™ DNA Polymerase (Thermo Scientific) according to standard protocol and PCR amplification products were sequenced on an ABI3730xl DNA Analyzer (Applied Biosystems), using the Big-Dye Terminator v3.1 kit and analyzed with Sequencher 5.0 software (Gene Codes Corporation). DNA mutation numbering was based on cDNA reference sequence (NM_052843 and NM_001458.4), taking nucleotide +1 as the A of the ATG translation initiation codon. The mutation nomenclature used follows that described at http://www.hgvs.org./mutnomen/.

### Whole exome sequencing (WES)

WES on patients III:3 and IV:2 of the French family was performed at ATLAS Biolabs GmbH using SeqCap EZ Human Exome Library v2.0 (Roche NimbleGen) for DNA capture. The enriched DNA was sequenced with an Illumina HiSeq 2000 platform, 2 x 100 bp with 80–90 x coverage. Reads were aligned to the human genome reference GRCh37/hg19 with Burrows-Wheeler Aligner and duplicate reads were removed with Picard. The Genome Analysis Toolkit was used to realign the reads, recalibrate base quality scores and call variants. Variants were annotated using Annovar and only exonic variants with a frequency ≤ 1% in the 1000 Genomes or Exome Sequencing Project (ESP6500) databases were selected. From this screening, 1516 variants shared by III:3 and IV:2 were selected. From these 1516 variants, 12 variants were further selected as they were found in genes known to be causative of muscle diseases or in potential candidate genes [23]. Among these 12 variants, 10 were excluded because they were either synonymous substitutions, or represented unlikely pathogenic variants, or they were in genes unrelated to the phenotype of these patients. WES on 3 patients and one normal relative from the Bulgarian families [8] and on five unrelated normal controls was performed at Centro di Ricerca Interdipartimentale per le Biotecnologie Innovative (CRIBI), Padova, Italy. DNA libraries for WES were constructed following the standard protocol of the Ion AmpliSeq™ Exome RDY Kit (ThermoFisher Scientific). Starting from 100 ng of gDNA, a multiplex-PCR with 12 primer pools was performed in order to allow the amplification of 24,000 amplicons per pool, covering the exonic regions of the genomes (about 58 Mb). Further 9 μl of 100 pM barcoded libraries were amplified by emulsion PCR using a OneTouch2 instrument. Finally, the barcoded samples were loaded into Ion Proton P1 v3 chip and sequenced on the Ion Proton instrument using the Ion Proton HiQ Sequencing kit. The bioinformatic analysis was performed on the Ion Torrent Server and the Torrent SuiteTM was used to base-call and align the reads to the human reference genome (GRCh37/hg19). Alignment BAM files, Coverage Analysis statistics and Variant Analysis VCF files were produced. In particular, average base coverage depth was 91.06 for IV:3 in family 2701, 95.2 for III:2 in family 2703 and 92.23 for III:1 in family 2702, with a percent base reads on target of 94.8%, 95.01% and 94.82%, respectively. By default, the Variant calling was performed using the “Germ Line-High Stringency” algorithm. Data were analyzed by QueryOR web platform [24] and genetic variants were filtered for a global minor allele frequency ≤0.001, shared by the affected individuals but not present in the asymptomatic member nor in normal controls.

### Generation of GST expression vectors and constructs for *in vitro* translation

Human *OBSCN* cDNA (NM_052843) coding for Ig58/Ig59 was amplified from total RNA extracted from human skeletal muscle tissue using specific primers (forward primer: 5’- aagaacacggtggtgcggg– 3’; reverse primer: 5’- gaggcccagcagggtgagc-3’). The *OBSCN* cDNA containing the c.13330C>T variant was generated by PCR using primers designed to introduce the cytosine to thymine nucleotide change into the wild-type cDNA (forward primer: 5’- aacgcggcggtcTgggccggcgcacag– 3’; reverse primer: 5’- ctgtgcgccggcccAgaccgccgcgtt-3’) and sequenced using an ABI Prism 310 apparatus (Applied Biosystems). The amplified sequences were cloned into the vector pGEX (GE Healthcare) using the EcoRI-SalI sites to generate GST-obscurin Ig58/Ig59^WT^ and GST-obscurin-Ig58/Ig59^MUT^ fusion proteins. Human *TTN* cDNA (NM_001267550) was amplified from total RNA extracted from human skeletal muscle tissue using specific primers. The *TTN* cDNA containing the ZIg9/ZIg10 domains of titin was generated by PCR using the following primers (forward primer: 5’—gacaaagagaaacaacagaaa– 3’; reverse primer: 5’–atcttccctctgttgaatctc– 3’) and sequenced using an ABI Prism 310 apparatus (Applied Biosystems). The amplified sequences were cloned into the vector pGBK-T7 in frame with a myc tag epitope (Clontech) using the EcoRI-SalI sites.

### Protein expression and *in vitro* interaction studies

GST fusion proteins produced in Escherichia coli [BL21(DE3)] were induced at OD_600_ = 0.6 with 1 mM isopropyl β–D-thiogalactopyranoside (IPTG) for 3 hrs at 30°C. Cells were harvested by centrifugation at 4000 x g for 10 min at 4°C. The pellet was resuspended in cold buffer containing Phosphate Buffer Saline (PBS), 1% Triton X-100, 20 mM EDTA and lysed by sonication on ice. The soluble fraction was obtained by centrifugation at 13200 x g for 15 min at 4°C. The fusion proteins were immobilized by incubating 1 ml of the soluble fraction with 100 μl of glutathione-Sepharose 4B resin (GE Healthcare, Buckinghamshire, United Kingdom) for 10 min and washed three times with 1 ml of a buffer containing PBS and 1% Triton X-100 [25]. Beads were finally resuspended with an equal volume of PBS.

*In vitro* translation and transcription experiments were performed using the TNT Quick Coupled Reticulocyte Lysate System as described by the manufacturer (Promega). 5 μl of the translation reaction were incubated with 12 μg of GST fusion protein in a buffer containing 10 mM Tris-HCl pH 7.9, 150 mM NaCl, 0.5% NONIDET P-40, 1 mM DL-Dithiothreitol (DTT), 1 mM phenylmethylsulfonyl fluoride (PMSF) and protein inhibitor mixture for 1.5 hrs at 4°C. After incubation, the GST fusion protein complexes were washed three times with interaction buffer. Bound proteins were eluted by boiling in SDS-PAGE sample buffer and analyzed by SDS-PAGE.

### Western blot and densitometric analysis

Protein samples were separated by 10% SDS-PAGE. Proteins were transferred to nitrocellulose membrane (Amersham Protran 0.45 μm NC, GE Healthcare) using a transfer buffer containing 192 mM Glycine, 25 mM Tris, 0.01% SDS and 10% Methanol for 5 hours at 350 mA at 4°C. Protein transfer was evaluated by Ponceau S staining of nitrocellulose membranes. Filters were incubated with primary antibody (mouse anti-c-myc, Clontech-Zymed Laboratories Inc.), diluted in blocking buffer (5% non-fat dry milk, 50 mM Tris-HCl pH 7.4, 150 mM NaCl, 0.2% Tween-20) overnight at 4°C with agitation. Filters were washed three times with washing buffer (0.5% non-fat dry milk, 50 mM Tris-HCl pH 7.4, 150 mM NaCl, 0.2% Tween-20) for 10 min each, incubated with horseradish peroxidase-conjugated secondary antibody and detected using the ECL system (ECL Western Blot Detection Reagents, Promega) and high performance chemiluminescence films (Amesham Hyperfilm ECL, GE Healthcare).

Densitometric analysis was performed using the ImageJ software. Chemiluminescence films were scanned and images converted to grayscale. Single lanes were selected and analyzed to generate a plot profile and to measure the density of the bands obtained following interaction of *in vitro* translated titin ZIg9/ZIg10 with either GST- obscurin-Ig58/Ig59^WT^ or GST-obscurin-Ig58/Ig59^MUT^. Band density was normalized to the amount of GST proteins used in each *in vitro* interaction assay. To this aim, following protein transfer and staining with Ponceau S, nitrocellulose membranes were scanned and images converted to grayscale. Single lanes were selected and analysed to measure the band density of GST-fusion proteins. Data were expressed as normalized band intensity using the GST- obscurin-Ig58/Ig59^WT^ interaction values as reference.

### NMR preparation and data collection

All chemicals were ACS grade or higher and were purchased from Fisher Scientific unless otherwise specified. Recombinant ^15^N, ^15^N-^13^C, and unlabeled proteins were purified after overexpression in BL21 cells using a pET24a vector system (Novagen, San Diego CA) in a manner similar to [26]. For purification, all proteins were passed over a G75-sepharose size exclusion column, and all eluted as monomers. All NMR experiments were collected on a 600 MHz Bruker Avance II spectrometer equipped with a TXI room temperature 5 mm probe with z-axis pulse field gradient coils. For the Ig59 structure, all NMR samples were collected at 25°C in 20 mM Tris pH 7.5, 20 mM NaCl, 0.35 mM NaN_3_, and 0.5–2.5 mM protein with 10% D_2_O. Titrations of unlabelled titin into obscurin were conducted with 0.5 mM of 15N-labeled obscurin Ig58/Ig59 and 0, 0.1, and 0.25 mM of unlabelled titin ZIg9/ZIg10 in 20 mM Tris pH 7.5, 20 mM NaCl, 0.35 mM NaN_3_ at 37°C. All 2D and 3D NMR experiments were collected as previously described [27]. Further description of NMR collection is provided in the Supplemental Information (S1 Text). Chemical shifts for Ig59 have been deposited in the Biological Magnetic Resonance Bank (BMRB) under accession number 26593.

### Structure calculation

Interproton distance constraints were derived from 3D NOESY experiments (^15^N-edited and ^13^C-edited 3D NOESY) as described previously. Dihedral constraints Ψ ± 20° and ψ ± 15° for α-helix and Ψ ± 40° and ψ ± 40° for ß-sheet were included based on TALOS+ and the chemical shift index of ^1^Hα and ^13^Cα atoms [27]. Residual dipolar coupling data was included based on splitting values from an IPAP experiment, as previously described [27]. More information about the structure calculations can be found in the supplemental information (S1 Text). The final 20 structures were selected (from 200) based on lowest Q-values and lowest root mean squared deviation (RMSD) from the average, and were of high quality based on the statistical criteria listed in S2 Table. The overall backbone RMSD of ordered heavy atoms is 0.609Å. With the exception of Arg 4430, and Arg 4508, every backbone H-N bond and most sidechain C-H shifts are visible in these NMR experiments. H-N residual dipolar coupling experiment values independently verify the validity of this structure, and result in a Q-factor of 0.25 ±0.02. The coordinates of the human obscurin Ig59 structure have been deposited in the Protein Data Bank (2N56).

The model of Ig58/Ig59 was calculated in the same fashion, using NOEs derived from ^15^N and ^15^N, ^13^C-labeled Ig58/Ig59 samples. However, since no interdomain NOEs were present, this dual domain system should be considered a model for illustration purposes only, and is not a high-resolution structure.

### Crystallization and X-ray diffraction

The hanging drop method with 17% tacsimate, 0.1M HEPES pH 7.5, 4% PEG3350, and 10 mg/ml protein was used to obtain Ig59 crystals. Crystals were harvested and frozen in liquid nitrogen after one week using a glucose cryoprotectant and crystallographic reflections were collected at the Structural Biology Center beamline 19-ID-D at the Advanced Photon Source, Argonne National Laboratory. HKL2000 data processing calculated the unit cell to be P 31 2 1.

### Structure refinement

Crystal diffraction phasing was determined in PHENIX ver 1.72.2–869 via molecular replacement using PDB from accession numbers 2YZ8 and 4RSV. The resulting structure was refined using the program phenix-refine. COOT was used to manually rebuild the structure in iterative rounds of rebuilding and refinement in PHENIX refine, resulting in a 1.18 Å resolution structure with an Rfree value of 0.185. More refinement statistics are given in S3 Table. The coordinates of Ig59 have been deposited in the PDB under accession number 5TZM.

### Molecular dynamics modelling

Coordinates corresponding to the crystal structure of Ig59 were allowed to equilibrate for 10 ns using YASARA 12.7.16, at 310K, 150 mmol/L NaCl, pH 7.4, using the Amber ff03 forcefield in a simulation cell with periodic boundaries until the backbone RMSD no longer changed significantly [28]. Simulations were run with a timestep of 1.25 fs with the temperature adjusted using a Berendsen thermostat as described by Krieger et al. [29]. Once finished, the p.Arg4444Trp mutation was introduced to the simulation using the”swap” function in YASARA. This mutated structure was then once again allowed to equilibrate for 10 ns using the same parameters as for the wild-type structure, and the simulation was terminated when the RMSD stabilized. YASARA and Pymol were used to visualize the structures.

### Circular dichroism (CD)

All CD experiments were conducted in a 1 mm pathlength cuvette at 5 μM protein in 20 mM Tris buffer, pH 7.5 and 100 mM NaCl. Samples were measured in triplicate on a Jasco J-810 spectrophotometer. Spectra were collected at 5°C intervals from 20°C to 85°C, with a 5 minute equilibration time after each temperature step.

### Surface plasmon resonance (SPR)

Surface plasmon resonance experiments were performed using a Nicoya Lifesciences Open SPR system equipped with an Au-GST chip. GST-linked titin ZIg9/ZIg10 was immobilized using standard procedures. Immobilization was monitored via absorbance change in a standard solution of filtered buffer (20 mM Tris pH 7.5, 50 mM NaCl) for 5 minutes at a flow rate of 20 μl/min. The chip was washed in 2 M MgCl_2_ to remove impurities. To collect kinetic binding data, WT or mutant obscurin Ig58/Ig59 in matched buffer at 27°C was injected to the flow cell at a flow rate of 50 μl/min and allowed to associate and dissociate for 120 and 480 s, respectively. The surface was regenerated with 2 M MgCl_2_ after each run. Data were collected at a rate of 5 Hz. Data were fit to a 1:1 interaction model using the analysis software TraceDrawer.

## Results

### Clinical findings

**Fig 1:**
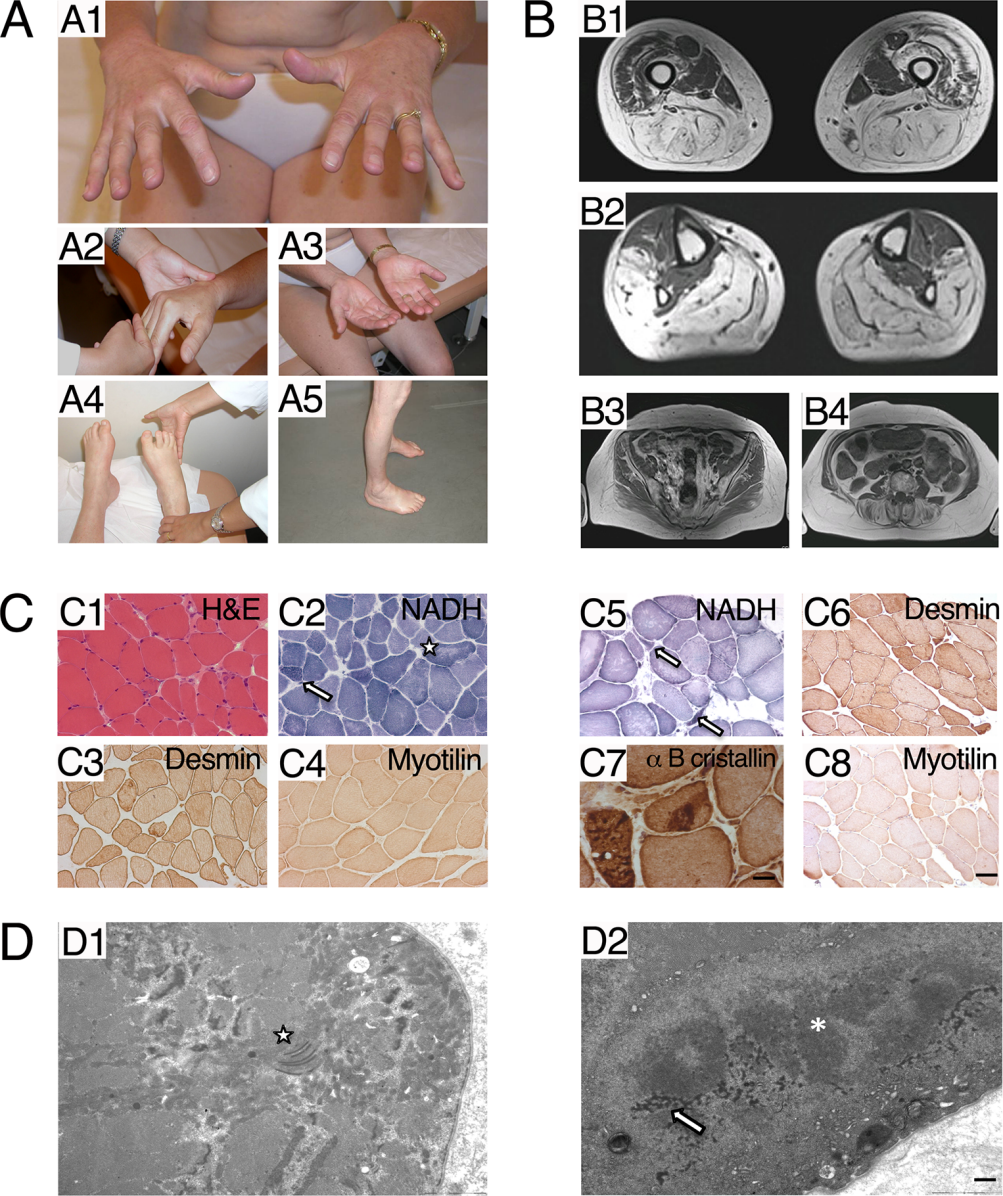
Clinical data. **A.** Clinical data of the proband (III:3). A1, A2: Hand extensors weakness. A3: Palmar atrophy, mainly in thenar muscles. A4: Peroneus lateralis, markedly affected, as all legs muscles except for tibialis anterior and posterior muscles. A5: Leg atrophy. **B.** MRI of the proband. B1: Thigh. Hamstrings and adductors severely involved; quadriceps partially spared, except right vastus lateralis and intermedialis. B2: Legs. Peroneal lateralis severely affected, as all muscles except tibialis anterior and tibialis posterior, B3: Pelvic muscles spared in comparison with femoro-distal muscles. **C.** Morphological studies. C1-C4 Radialis muscle biopsy of proband (III:3). C1. Haematoxylin & Eosin staining: presence of some nuclear internalization and fiber size variation. C2 NADH: lobulated fibers are indicated by an arrow; uneven staining of the intermyofibrillar network in some fibers is indicated by a star. C3 desmin: mild diffuse desmin surcharge in several fibers C4 myotilin: normal staining. C5-C8 Peroneal muscle biopsy from the proband’s son (IV:2). C5 NADH: atrophic lobulated fibers are indicated by an arrow. C6 desmin: mild diffuse desmin surcharge in several fibers. C7 alpha B cristallin: presence of dense protein aggregates. C4 myotilin: normal staining (Scale bar = 20 μm for all images and 8 μm only in C7). **D.** Ultrastructural studies: Radialis muscle biopsy of proband (III:3), EM analysis. D1: presence of numerous abnormal mitochondria harbouring dotty or paracrystallin inclusions, in proximity of the star. D2: presence of a cytoplasmic protein aggregate composed by dark osmiophilic granulo-filamentous material corresponding to desmin, indicated by an arrow, and filamentous material indicated by an asterisk (Scale bar = 1 μm).

The proband (III:3) is a 61-year-old female presenting a progressive distal dystrophy since around age of 30, affecting successively the right hand and progressing slowly to include both distal upper and lower limbs. At age 45, difficulties in standing up from a chair and a steppage gait appeared. At age 51, ambulation was not limited, but examination showed foot drop, more pronounced in the right side, inability to stand on tip toes and heels, and waddling gait. There was a severe weakness of hand finger extensors (Fig 1A1 and 1A2), foot evertors (in contrast to normal finger flexors and tibialis anterior, respectively), toes extensors, calf muscles. Thigh adductors and hamstrings were also severely affected, but proximal scapular, brachial, and antebrachial involvement was not detected. Hand palmar muscles (mainly thenar ones) (Fig 1A3), calves and tibial muscles were atrophic (Fig 1A4 and 1A5). In addition to the worsening myopathy, a progressive cervical myelitis of unknown aetiology appeared at age 52, manifesting as a sensory defect mainly in left lower limb and spreading from foot to hip, causing severe pain and impaired balance. At age 61, she could walk slowly a few hundreds meters with two aids. The previously described clinical selective pattern was found with severe wasting of hand finger extensors, toes extensors, peroneus, posterior calf muscles, hamstrings which was contrasting with partially spared hand finger flexors, tibialis anterior, quadriceps. Involvement of iliopsoas, glutei medii was moderate. Scapular girdle, brachial, antebrachial, axial, facial and oculo-bulbar muscles were spared. Quantitative measurements are detailed in Table 1. A sensory defect due to the myelopathy was found in both distal and proximal lower limbs (left > right). Creatine kinase (CK) levels were normal. Electromyogram (EMG) showed myopathic changes distally in the upper limbs and both proximally and distally in the lower limbs. Cardiac evaluation (ECG and echocardiography) and spirometry were normal. Spinal cord MRI performed at age 53 revealed a posterior hyperdense signal from C2 to C7. Brain MRI and CSF were normal. The proband’s son (IV:2) noticed first symptoms (fatigability in walking and difficulties in standing on tiptoe) at age 14. He has been unable to run since the age of 16 years. At age 33, walking distance was around 1000 meters, without aid. He had marked weakness and atrophy in the calf muscles and minor weakness in the interossei and finger extension, but otherwise normal muscle strength in the proximal lower limbs and in the upper limbs (Table 1). No facial or bulbar weakness was present. CK levels, spirometry and cardiac evaluation were normal. EMG showed myopathic changes.

**Table 1:** Clinical data of the patients.

Case Age Sex	Symptom onset (age in years)	Course	Current status: (age) functional ability	Current status Muscle weakness	Current status Muscle atrophy	CPK/ EMG / Cardiac echography/ EKG/ Vital capacity
Case I Proband (III:3) **61y**	Right hand atrophy / weakness (finger extensors) **(≈ 30y)**	Progressive worsening **Around 30y**: both hands wasting, inability to stand on heels and tiptoes, 35 y difficulties climbing stairs. 46 y Gowers, difficulties standing from a chair, running inability. Increased weakness of foot evertors, hand finger extensors **52y**: cervical myelitis manifesting by left foot extending to whole limb after 6 months. sensory defect without identified cause (Spine MRI T2 sequence hypersignal from C2 to C7) (normal Brain MRI, normal CSF fluid) Increased loss of balance and cordonnal pains **53 y**: fallings (weakness and balance impairment) **59 y**: walk with bilateral aid	**(61y)** Walking distance: few hundreds meters with aid Fallings, four to five times monthly right foot elevator Difficult walk with steppage and tendency to internal rotation of feet 10 m in 15 s with a cane. Inability to stand on heels and tiptoes Rising from chair 9 times in 60 s with two hands Thigh crossing: difficult Arm elevation: normal Painful spinal posterior column syndrome (myelitis)	**Lower limbs** Toes extensors 2, except big toe, 1, Peroneus lateralis 1, Tibialis anterior 4, Post legs 2+ Hamstrings 2, Quadriceps 5- Adductors 2+, Glutei medii 4+, Iliopsoas 3 + **Upper limbs** Finger extensors 3-, index more affected: right index 2, left 2+ Finger flexors 4+ wrist extensors 4 scapular and brachial 5 **Axial,facial, oculobulbar** normal in addition: hypoesthesia of whole left lower limb due to myelitis	Legs (both compartments) Hand: palmar, mosly thenar region	CK normal EMG: myogenic Distal in Lower limbs, Distal + proximal in lower limbs Nerve: normal No decrement Normal echography and EKG VC: 85%
Case II Son of case I (IV:2) 33 y	Difficulties standing on tiptoes and heels fatigability in walking (14y)	Mild progression At 16 y, interruption of sport at school and inability to run.	**(33y)** Walking distance: 1000 m without aid No fallings Climbing stairs with banister 10 m: 12s Rising from a chair: 24 times in 60 s without aid Arm elevation: normal	**Lower limbs** Peroneus 3-, Tibialis anterior 3+ Toes extensors between 4, and 2 (big toe) Post legs 2 Pelvic and femoral muscles 5 **Upper limbs** Finger extensors 4+, Finger flexors 5 wrist extensors 5 scapular and brachial 5 **Axial, facial, oculobulbar** normal	Legs, mainly posterior compartment No atrophy of upper limbs	CK normal EMG: diffuse myogenic pattern Nerve: normal Normal echography and EKG VC: 86%

Abbreviations: CSF (Cerebral spinal fluid); EKG (Electrocardiogram); EMG (Electromyogram); VC (Vital capacity); CK (Creatine Kinase)

Muscle imaging performed in the mother at the age of 51 showed severe fatty degenerative changes in almost all lower leg muscles, except for tibialis anterior, posterior and toes extensor muscles, and quadriceps that were much less affected than hamstrings and adductors. The forearm extensor muscles showed reduced volume. At age of 61 years, the main pattern was found, but vastus lateralis and vastus internmedius fatty degeneration was more marked, particularly in right side. Rectus femoris and vastus medialis remained spared (Fig 1B1, 1B2 and 1B3). The changes in Computed Tomography (CT) scan performed at age 33 in the son were milder but he also had severe fatty replacement in the calf muscles and less severely in the lateral compartment peroneal muscles. All other muscles were intact.

The radialis muscle biopsy from the proband (III:3) performed at 51 years showed some nuclear internalization, fiber size variation (Fig 1C1), and type 1 predominance. Oxidative histoenzymatic reactions disclosed few lobulated fibers and uneven staining of the intermyofibrillar network (Fig 1C2). A peroneal muscle biopsy of the son (IV:2) at the age of 33 years showed marked fiber size variability with the presence of some atrophic angulated fibers (Fig 1C5). Few lobulated fibers were also present. Immunohistochemistry performed in both muscle biopsies revealed some desmin surcharge in both patients (Fig 1C3 and 1C6). In contrast only patient IV:2 harboured alpha B crystallin immunoreactive aggregates (Fig 1C7). Myotilin immunostaining did not show specific alterations (Fig 1C4 and 1C8). Ultrastructural studies in proband (III:3) showed myofibrillar disorganisations resembling targetoid structures in a small percentage of the fibres (not shown). Abnormal mitochondria with some paracristalline inclusions (Fig 1D1 indicated by a star) and dark dots were observed in numerous fibres. Focal disintegration of myofibrils, dark osmiophilic granulo-filamentous material corresponding to desmin (Fig 1D2 indicated by an arrow) and filamentous protein aggregates (Fig 1D2 indicated by an asterisk) were observed in patient IV:2. The clinical findings of the patients from Bulgarian families with distal myopathy with upper limb predominance have been previously described [8].

### Molecular genetics

**Fig 2:**
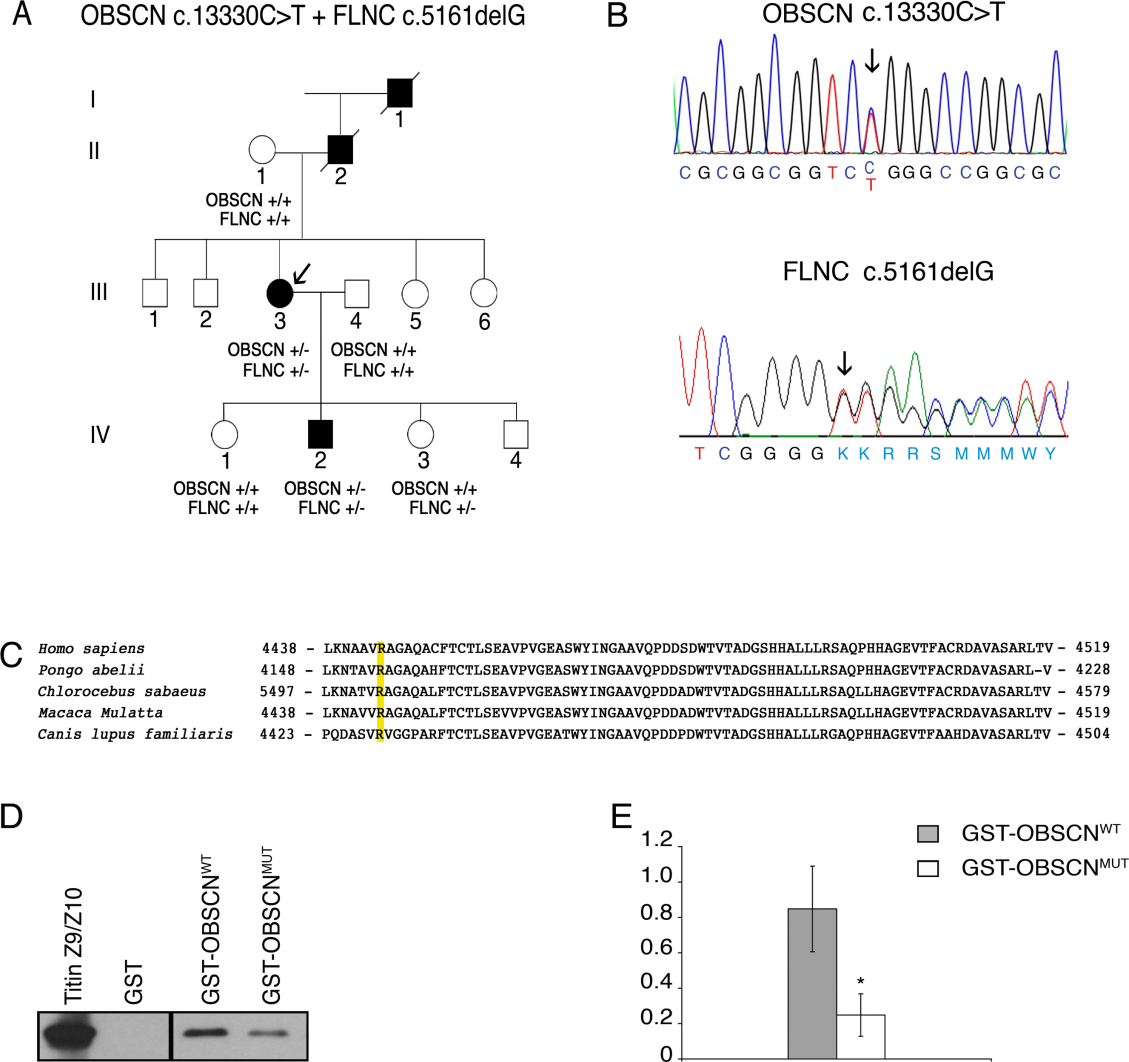
Genetic analysis of *OBSCN* c.13330 C>T and *FLNC* c.5161delG mutations. **A.** Family pedigree. Black filled symbols represent affected family members. The genotype of individuals is shown as follows: +/+ wild type, OBSCN +/- heterozygous for the c.13330 C>T mutation, FLNC +/- heterozygous for the c.5161delG mutation. Arrow indicates the proband. **B.** Electropherogram of the *OBSCN* gene sequence (upper panel) and of the *FLNC* gene sequence (lower panel). **C**. Phylogenetic alignment of the *OBSCN* orthologs. Sequences represent the Ig59 domains of obscurin in distinct mammalian species. The arginine in position 4444 in the human *OBSCN* sequence is highlighted in yellow and is conserved in mammalian species. **D.** Interaction between titin domains ZIg9/ZIg10 and obscurin domains Ig58/Ig59. *In vitro* transcribed and translated myc-tagged titin domains ZIg9/ZIg10 were used in pull-down experiments with GST-fusion proteins containing either wild type Ig58/Ig59 domains (GST-obscurin-Ig58/Ig59^WT^) or R4444W mutated Ig58/Ig59 domains (GST-obscurin-Ig58/Ig59^MUT^). Proteins were separated by SDS-PAGE, transferred to membranes and detected by mouse anti-myc antibodies. **E.** Quantification of GST pull-down efficiency. Protein bands intensities of myc-tagged ZIg9/ZIg10 titin domains precipitated by either GST-obscurin-Ig58/Ig59^WT^ or GST-obscurin-Ig58/Ig59^MUT^ GST-fusion proteins were evaluated by densitometric analysis and normalized on GST fusion proteins content. Data represent means values ± standard deviation of five independent experiments in triplicate. * p < 0.01 following t-test stastistical analysis.

Based on the evidence that mutations in the M10 domain of TTN, which binds the Ig1 of obscurin, are found in patients with muscular dystrophy we sequenced the *OBSCN* exons encoding the titin-binding region at both M-band and Z-disk of patients with a clinical history of molecularly undiagnosed distal dystrophy. This analysis revealed the presence of a heterozygous missense variant c.13330C>T in exon 51 predicting an amino acid change from arginine to tryptophan (p.Arg4444Trp) in the Ig59 of obscurin (according to sequence NP_443075) (Fig 2B) in the proband of a French family (Fig 2A individual III:3). Sequence analysis of *OBSCN* exons 2, 50, 51 and 52 performed on 110 unrelated probands with different forms of muscular dystrophy did not identify mutations in these sites. The c.13330C>T substitution is reported as variant rs369758958, with an allele frequency of 1/8325 in the European/American population (1000 genomes database and ESP6500). The same variant was described in 2 South Asian as well as in 1 Latino and 1 European (non Finnish) individual, with a MAF of 0.00003770, according to ExAC genome database. An additional variant c.13331G>A (rs372781730) resulting in an amino acid change from arginine to glutamine (p.Arg4444Gln) was described in 1 Latino and 1 South Asian individual, with MAF of 0.00001881 (ExAC genome database). The p.Arg4444Gln variant affects an amino acid residue conserved across mammals (Genomic Evolutionary Rate Profiling (GERP) score 2.96), and is predicted to be a possibly damaging substitution (0.920 according to PolyPhen) in a region of obscurin involved in titin binding (Fig 2C). The variant fully segregated with the disease phenotype in the family analyzed, as was detected in the proband and in her affected son but not in other healthy relatives (Fig 2A).

WES analysis, performed with the DNA from the proband (III:3), in addition to confirm the heterozygous missense variant in *OBSCN*, also identified a novel frameshift mutation in *FLNC* (NM_001458.4). This was a deletion of a single base in exon 30 (c.5161delG) resulting in a frameshift and a premature stop codon (p.Gly1722ValfsTer61, Fig 2B). This mutation is not present in the 1000 genomes, ESP6500 or ExAC databases. Sanger sequencing for *FLNC* exon 30 was performed on II:1, III:3, III:4, IV:1, IV:2 and IV:3. The *FLNC* variant c.5161delG was present in the two affected individuals (III:3, IV:2) but also in one healthy individual (IV:3). This deletion is located adjacent to the previously reported c.5160delC deletion, which is linked to distal myopathy with upper limb predominance in three Bulgarian families [8]. To investigate if the *OBSCN* variant identified in the French family was present also in the Bulgarian families with recurrence of the *FLNC* c.5160delC frameshift, we initially performed Sanger sequencing of exons 2, 50, 51 and 52 of *OBSCN* in 12 individuals from the three related Bulgarian families (3 unaffected non-carriers of the *FLNC* c.5160delC (2701:IV.1, 2702:III.3; 2703:IV.3), 4 asymptomatic carriers of the *FLNC* c.5160delC (2701:IV.16; 2701:V.1; 2701:V.2; 2702:IV.3) and 5 individuals with distal dystrophy carrying the *FLNC* c.5160delC (2701:IV.3; 2701:IV.10; 2702:III.1; 2702:IV.1; 2703:III.2). Since this initial screening did not identify any variant in the selected exons of *OBSCN*, WES analysis was performed in three affected *FLNC* mutation carriers, in one non-affected carrier relative from the Bulgarian families (patients IV:3 of family 2701, III:1 of family 2702, III:2 of family 2703 and individual IV:1 of family 2701, see supplemental Figure_e-1 in reference [8]), and in five unrelated normal controls. Data were analyzed by QueryOR web platform [24] and genetic variants were filtered for a global minor allele frequency ≤0.001, shared by the affected individuals but not present in the asymptomatic member nor in normal controls. This analysis identified no mutation (missense, nonsense, indel) shared by the affected patients.

### Structural studies of the Ig59 domain of obscurin

**Fig 3:**
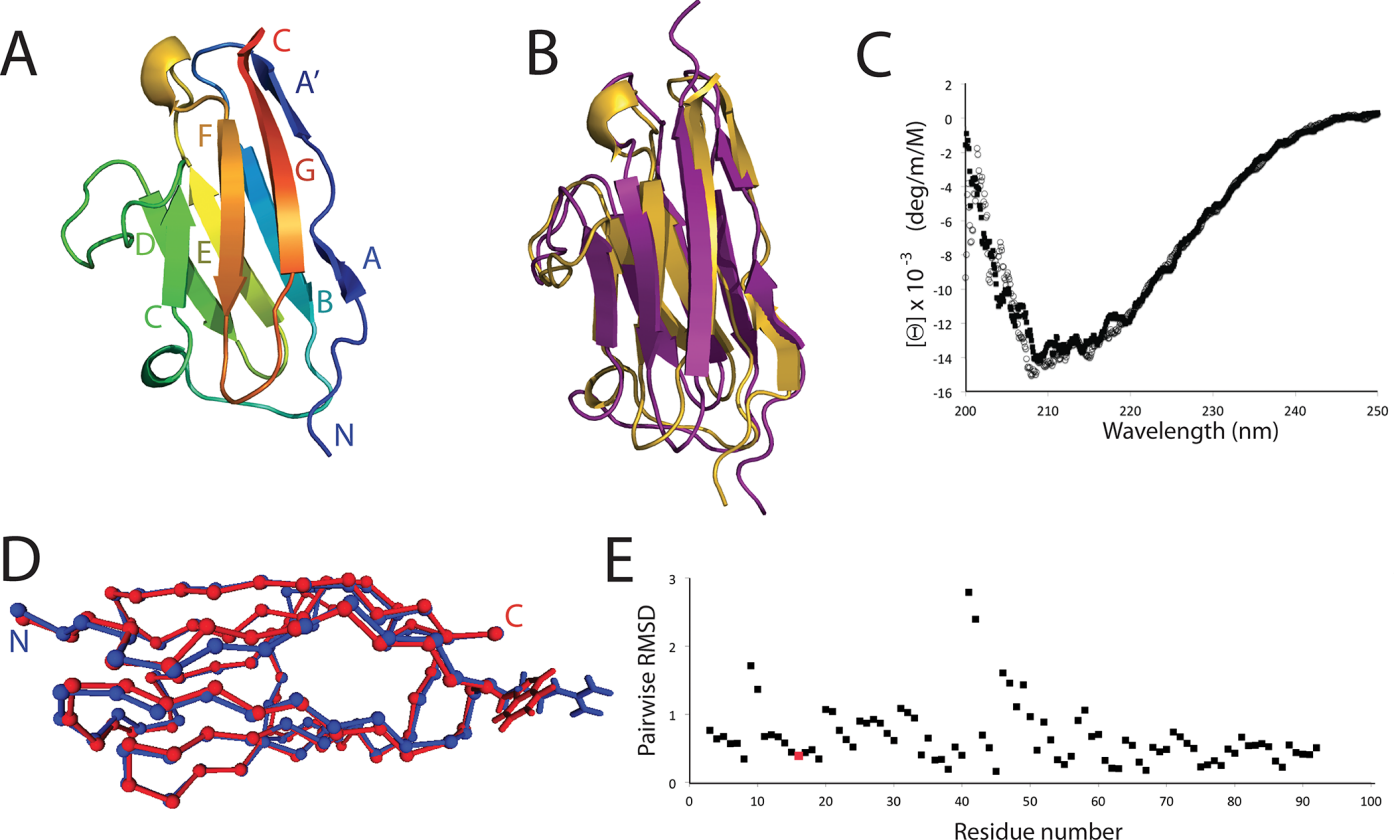
Structure and analysis of human obscurin Ig59. **A**. Cartoon of the Ig59 crystal structure, showing the typical Ig-like fold. **B**. Comparison between the lowest RMSD Ig59 NMR structure and the X-ray structure. **C**. CD plot of WT obscurin Ig58-59 (black squares) and p.Arg4444Trp (open circles). **D**. MD simulated average models of WT (blue) and p.Arg4444Trp (red). Ca position. The side chains for Arg4444/Trp4444 are shown. E. RMSD vs residue number comparison of the mutant model to the wild-type model. The Arg4444Trp site is colored in red.

Next, we wanted to verify whether the p.Arg4444Trp OBSCN variant affects the functional properties of the Ig59 domain such that it might act in increasing penetrance of the *FLNC* mutation. In order to begin defining the molecular mechanism of the described phenotype, we investigated how the normal and mutated obscurin Ig59 domain interacts with its binding partner TTN. We first solved the high-resolution structure of Ig59 (residues 4428–4521) [30]. It should be noted that obscurin Ig48 and Ig49 in reference 30 correspond to the current Ig58 and Ig59, according to a more recent nomenclature, as from sequence NM_052843). Human obscurin Ig59 was isolated based on the original sequence alignment (CAC44768.1) [13]. After purification, Ig59 was crystallized (Fig 3A, S2 Table). The resulting structure shows beta sheets extending from Glu 4436 to Lys 4439 (strand A), Ala 4441 to Arg 4444 (strand A’), Ala 4445 to Thr 4454 (strand B), Ser 4465 to Ile 4469 (strand C), Trp 4480 to Asp 4485 (stand D), His 4488 to Leu 4494 (strand E), Gly 4502 to Ala 4507 (stand F), and Ala 4513 to Leu 4520 (strand G). Overall, the structure forms a typical Ig-like fold [31]. Independently, the NMR solution structure of human obscurin Ig59 was solved with greater than 12 restraints per residue (Fig 3B, S3 Table, S1 Fig). The best NMR structure, as defined as that with the lowest RMSD, and X-ray structure are highly similar to each other, with a pairwise RMSD of 1.609 Å (Fig 3B).

The p.Arg4444Trp mutation could affect the obscurin-titin interaction in one of two ways: it may unfold the entire Ig59 domain, or it may maintain the fold and simply perturb the binding event. Circular dichroism (CD) experiments on the normal and mutant versions of the Ig59 domain are virtually identical, suggesting that the mutation does not globally disrupt the overall Ig-like architecture (Fig 3C). Additionally, CD unfolding experiments on WT and mutant obscurin showed no significant change in overall stability (S2 Fig). Supporting this, molecular dynamics simulations of the WT and mutant domain indicated that the region surrounding the mutation was almost unchanged (Fig 3D and 3E). HSQC spectra between WT and mutant Ig59 domains (S3–S5 Figs) showed that the vast majority of backbone chemical shifts remain unchanged in the mutant. The largest chemical shift changes in the mutant version correspond to residues directly neighbouring the Arg4444Trp position in three-dimensional space. These changes are quite small (several dozen Hz), and likely reflect local changes to the Ig59 chemical shift environment. These data, combined with the CD data and MD simulations, led us to conclude that this mutation does not significantly change the Ig59 structure or stability. Therefore, the p.Arg4444Trp variant likely directly alters the obscurin-titin interface.

The Ig59 domain is only a part of the titin binding region; both obscurin Ig58 and Ig59 domains and both titin ZIg9 and ZIg10 domains are necessary for this interaction at the Z-disk [30]. Therefore, to obtain a more complete picture of this interaction, we next studied whether the inclusion of Ig58 affects the Ig59 structure significantly. The structure and assignments of Ig58 were recently published [32]. These data, along with the structural studies of Ig59 in this current paper, provide a platform to examine the NMR spectra of a tandem obscurin Ig58/Ig59 construct. The HSQC of the dual domain system overlays almost exactly as the sum of the Ig58 and Ig59 spectra. This attribute allowed almost complete HSQC assignment of this larger system.

In mapping the HSQC chemical shifts onto a model of both domains, the largest chemical shift changes between the individual domains and the dual domain system are those residues in and surrounding the short ‘Gly-Trp’ interdomain linker, although none of the chemical shifts are large (S6–S8 Figs). Thus, obscurin Ig58 and Ig59 do not significantly interact with each other, except possibly very minimally in regions surrounding the short linker. This point was further verified from NOE data, where we could find no instances of inter-domain interactions (S9 Fig).

**Fig 4:**
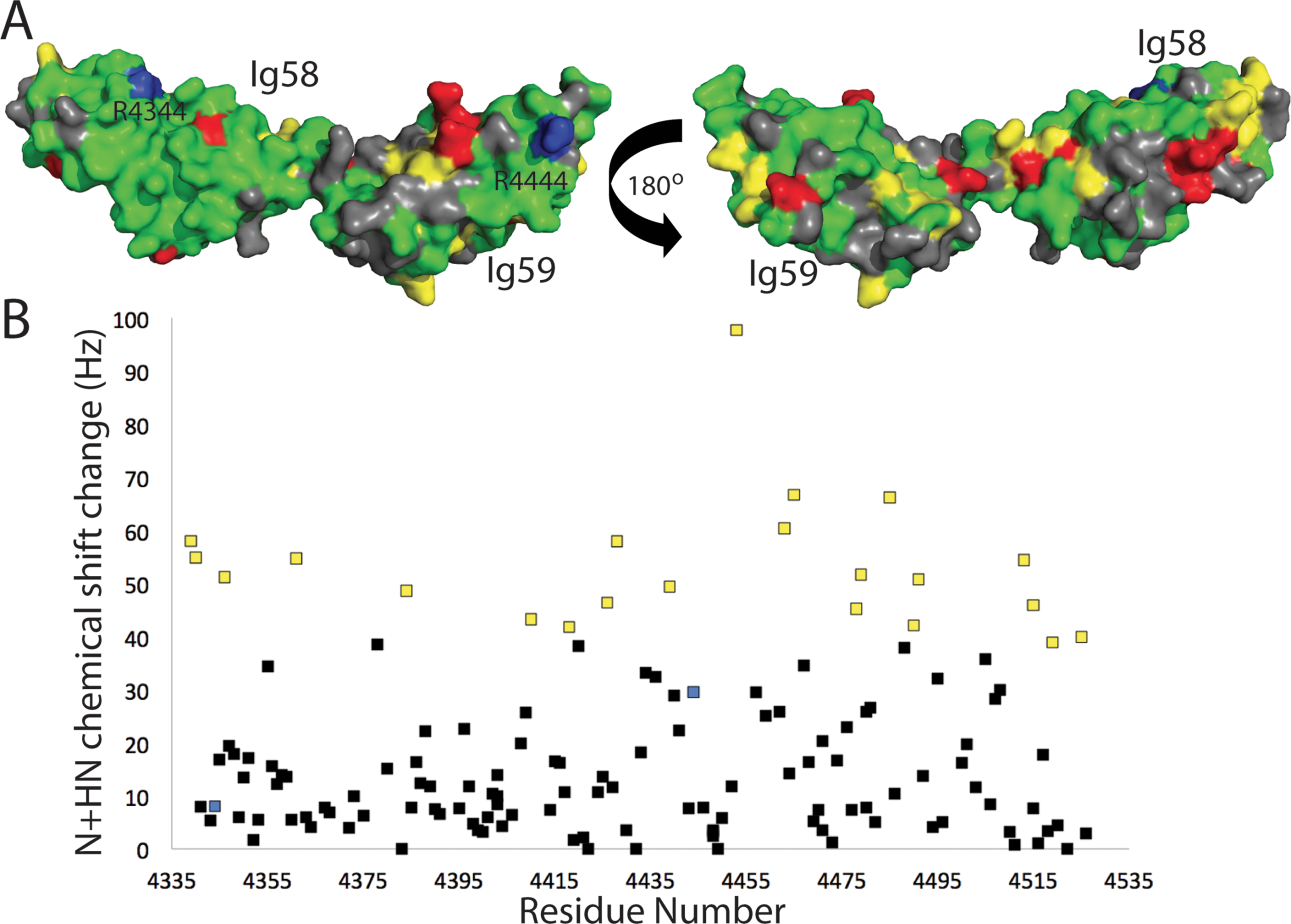
Both domains of obscurin Ig58/Ig59 are involved in binding titin ZIg9/ZIg10. HSQC chemical shift changes of Ig58/Ig59 upon the addition of 0.5x human titin ZIg9/ZIg10, mapped onto a model of obscurin Ig58/Ig59 (A) or by residue number (B). Gray denotes residues with no data; green denotes residues with no significant chemical shift changes; yellow denotes residues chemical shift changes greater than 2x standard deviation; red denotes residues experiencing a large drop in peak intensity of greater than 2.5x the average intensity. The two obscurin mutations linked to muscle disease, Arg4344 and Arg4444, are labeled and colored blue.

We next titrated unlabeled titin ZIg9/ZIg10 into a sample of ^15^N-labeled obscurin Ig58/Ig59 and monitored the chemical shift perturbations. By observing which residues experience the largest chemical shift changes, we can map the titin-obscurin binding interface. Increasing amounts of titin ZIg9/ZIg10 led to gradual small chemical shift changes in most assigned Ig58/I59 HSQC peaks. However, at a ratio of more than 0.5x titin:obscurin, most obscurin Ig58/Ig59 peaks become too weak to be detected, even with days-long HSQC collection times or TROSY experiments (S10 Fig). This was expected: the entire titin-obscurin complex approaches 55 kDa, and a byproduct of this size is extremely fast relaxation times. Therefore, we mapped the chemical shift changes at the 0.5x titin ratio. These data show significantly higher than average chemical shift changes in 21 residues, in both the Ig58 and the Ig59 domains (Fig 4, yellow). 16 peaks do not significantly move, but instead have significantly weaker intensities. We interpret these as probable slow- or intermediately-exchanging peaks (Fig 4, red). The resulting data, when mapped onto the Ig58/Ig59 model, show that two patches on a single face of Ig58, the interdomain linker, and several small Ig59 regions are in significantly different molecular environments upon the addition of titin ZIg9/ZIg10. These data suggest a global interaction with ZIg9/ZIg10, involving both obscurin domains and possibly also a reorientation of the linker region. Interestingly, more than one fourth of the coloured residues (11 out of 37) are charged. This may suggest that the obscurin/titin interaction is driven through electrostatic interactions. Neither the mutation described here (Arg4444Trp) nor the mutation described in a previous paper (Arg4344Gln) themselves experience large chemical shift perturbation [17]. However, both are near regions that do experience significant changes.

### The p.Arg4444Trp variant reduces the interaction between obscurin Ig58/Ig59 and titin ZIg9/ZIg10 domains

To directly evaluate whether the p.Arg4444Trp variant alters the interaction between obscurin Ig58/Ig59 and titin ZIg9/ZIg10, we performed GST-pull down assays with either immobilized GST- obscurin-Ig58/Ig59^WT^ or GST-obscurin-Ig58/Ig59^MUT^ mixed with *in vitro* translated titin ZIg9/ZIg10 domains, cloned in frame with a myc-tag epitope. Densitometric quantification of the binding between the ZIg9/ZIg10 domains of TTN and the Ig58/Ig59 domains of obscurin showed that the bound fraction of GST-Ig58/ Ig59^WT^ protein was about two fold higher than that of the GST-Ig58/Ig59^MUT^, indicating that the OBSCN p.Arg4444Trp variant significantly weakens the titin- Ig58/Ig59 interaction (Fig 2D and 2E). To further verify the effect of the OBSCN p.Arg4444Trp variant on the interaction with titin, we performed SPR experiments to determine the kinetics of binding of mutant and wild type Ig58/Ig59 domains to the ZIg9/ZIg10 domains of titin. These experiments revealed a ~15-fold weaker interaction between titin and the mutant obscurin Ig58/Ig59 region, with a kD of 1.6 μM and 108 nM for the mutant and the wild type Ig58/Ig59 domains, respectively (S11 Fig).

## Discussion

Screening of families with molecularly undetermined distal myopathy led to the identification of a c.5161delG frameshift mutation in *FLNC* in two members of a French family affected by distal myopathy and in one healthy relative. In addition, in these two French patients, but not in the healthy relative, we also identified a c.13330C>T (p.Arg4444Trp) variant in *OBSCN*. The protein product of *FLNC*, Filamin C, is an actin cross-linking protein localized at the Z-disk, miotendineous junction and sarcolemma in skeletal muscle [33]. Mutaions in *FLNC* are known to cause adult-onset myofibrillar myopathy and distal muscular dystrophy [9, 33–38]. In myofibrillar myopathies, mutations in *FLNC* were reported to destabilize muscle tissue homeostasis via the formation of protein aggregates [37]. On the contrary, missense mutations in the N-terminal actin binding domain of FLNC [7] and the frameshift deletion c.5160delC have been identified in distal myopathies without myofibrillar aggregates [8]. The c.5161delG mutation reported here is positioned only one nucleotide away from the c.51610delC *FLNC* mutation detected in three Bulgarian families with distal myopathy [8]. In those Bulgarian families, the c.51610delC *FLNC* mutation was shown to result in haploinsufficiency and to have an incomplete penetrance, as indicated by the presence of several healthy asymptomatic carriers. Although the small size of the French family represents a limiting factor for comparing the phenotype of these patients with that of the Bulgarian families, some differences can be observed between these two groups of patients. Histological evaluation of muscle biopsies from the French and Bulgarian patients did not reveal the presence of myofibrillar aggregates or other significant differences. Early clinical manifestations were similar in the two groups, with initial involvement of upper limbs, and lower limb involvement upon disease progression. Differences between the two groups were observed with respect to the time of disease onset and progression. Disease onset in the Bulgarian families was typically observed in the adult age (average 41±11 years, range 20–57 years), while in the French IV:2 subject the first symptoms were noticed when he was 14 years old and he became unable to run at the age of 16 years. In addition, at the date of the manuscript publication, all but one affected Bulgarian subjects remained ambulatory, while the French proband III:3 is currently unable to walk and is confined to a wheelchair.

In distal myopathies with myofibrillar aggregates, the aggregates represent a sign of altered assembly of myofibrillar proteins. In contrast, the pathomechanisms of distal myopathy caused by *FLNC* frameshift mutations without myofibrillar aggregates are currently not known [8, 38, 39]. In the Bulgarian families, the c.51610delC *FLNC* mutation resulted in haploinsufficiency with absence of protein aggregates [8]. We have no direct evidence of haploinsufficiency for the *FLNC* c.5161delG mutation, but given the close localization to the c.51610delC *FLNC* mutation and the absence of myofibrillar aggregates in muscle biopsies, the two mutations are expected to share similar pathomechanisms. Experiments in C2C12 cell models and knockout mice indicated that loss of FLNC expression results in severe alteration in muscle development and viability [40]. Similar results, using fish models, link a lack of FLNC expression to skeletal muscle abnormalities, disorganization of the Z-disk and loss of fiber integrity during muscle contraction [41, 42]. Data on heterozygous knockout mice or fish models for FLNC did not show any obvious phenotype. However, these studies were performed in young animals, while distal myopathy due to FLNC haploinsufficiency in humans has a typical adult onset, and therefore would require studies performed on animals at older ages [8, 38]. Moreover, the pathophysiology of *FLNC* frameshift mutations in distal myopathy is further complicated by the evidence of reduced penetrance, as both affected and asymptomatic carriers can be detected.

Interestingly, in the French family, the two patients with distal muscular dystrophy, in addition to the *FLNC* frameshift, also carry a c.13330C>T (p.Arg4444Trp) *OBSCN* variant that is not present in the healthy members of the family. Extension of WES studies to representative individuals in the Bulgarian families did not reveal the presence of other pathogenic variants shared by these patients. Thus, the genetics behind the incomplete penetrance of the disease in the Bulgarian families remains unsolved.

The interaction between the obscurin Ig58/Ig59 and the titin ZIg9/ZIg10 is recognized as important for targeting obscurin to the Z-disk [13–16]. Mutations altering the obscurin/titin binding site at the level of the M-band have been identified in patients with TMD and LGMD2J [4,6,23]. Our results from GST pull-down and SPR experiments indicated that the OBSCN p.Arg4444Trp variant results in a significant decrease in binding between the Ig58/Ig59 domains and the titin ZIg9/ZIg10. The reduced affinity observed in our *in vitro* experiments may certainly decrease the stability of the interaction between obscurin and titin at the Z-disk.

A different *OBSCN* mutation (p. Arg4344Gln) in the Ig58 was identified in a patient with dilated cardiomyopathy and was also found to display reduced titin binding, again pointing to the obscurin/titin binding sites as crucial for muscle physiology [17]. Although the frequency of this mutation (MAF >0.01165) is higher than the threshold for a disease causing variant, recent results from experiments with a knock-in mouse model carrying the pArg4344Gln mutation indicated that these mice, at 1 year of age, develop tachycardia accompanied by premature ventricular contractions and, if subjected to pressure overload, can also develop a dilated cardiomyopathy-like phenotype [32]. However it is unlikely the p.Arg4344Gln and the p.Arg4444Trp work via the same molecular mechanism. The pArg4344Gln mutation seems to rely primarily on a gain-of-function mechanism where the mutant obscurin binds to phospholamban, a Ca^2+^ ATPase (SERCA) pump inhibitor [32]. This binding event causes SERCA misregulation that may explain development of the cardiomyopathy phenotype [32].

In conclusion, we identified a novel *FLNC* c.5161delG frameshift mutation and an *OBSCN* variant in two patients in a French family with distal muscular dystrophy. Interestingly, both OBSCN and FLNC are Z-disk associated proteins. Based on similarities with previously reported findings on the *FLNC* c.5160delC frameshift mutation identified in Bulgarian families with distal myopathy, we propose that the novel *FLNC* c.5161delG (p.Gly1722ValfsTer61) mutation is responsible for the distal myopathy observed in the French family. In the two French patients, but not in their healthy relatives, we also identified a c.13330C>T (p.Arg4444Trp) variant in *OBSCN*. No relatives carrying only the *OBSCN* variant were detected in the French family, so we cannot definitely evaluate whether the *OBSCN* variant alone might have a causative role in distal myopathy. However, even if the small size of the French family represents a limiting factor for evaluating the pathogenic relevance of the OBSCN p.Arg4444Trp variant, the earlier time of disease onset and the more severe progression, combined with molecular biology data showing that the p.Arg4444Trp mutation specifically results in a ~15-fold decrease in titin binding at the Z-disk, lead us to hypothesize that the *OBSCN* variant might contribute to promote the full expression of the myopathic phenotype in the patients carrying the *FLNC* c.5161delG mutation.

## Supporting information

S1 Text NMR collection details and NMR structural calculation details. (DOC)

S1 Fig NMR data used to determine the structure of human obscurin Ig59. A) Observed beta sheet interactions of Ig59 as seen in the 15N-edited NOESY. B) Example of NOESY data (in black) overlaid with TOCSY data (in red), showing self-peaks and cross-strand NOEs. C) CBCA(CO)NH (in red) and HNCACB (in black) experiments, showing an example of NMR backbone assignments. D) Example of residual dipolar coupling data showing isotropic (right) and anisotropic (left) examples of the H-N bond from A78.
(TIF)

S2 Fig Temperature unfolding of WT Ig58/Ig59 and the p.Arg4444Trp Ig58/Ig59. Circular Dichroism traces from 250–200 nm display a typical beta sheet-like trace, with a minimum at 217 nm. Upon heating, this peak widens (Rudloff et al., 2015). Thus, one read-out for Ig domain unfolding is CD signal at the shoulders of this 217 nm peak; as the peak widens with unfolding, this signal becomes more negative. Here, we measure the CD signal at 225 nm, using 10μM protein, 50 mM NaCl, 20 mM Tris pH 7.5 for WT (blue circle) and R444W (red square) in a 1 mm cuvette. The T_M_, as calculated by taking the first derivative of these data, show WT to unfolds at 58°C and the Arg4444Trp mutant to unfolds at 55°C.
(TIF)

S3 Fig Labeled HSQC of Ig59. Conditions are at 25 ^o^C, 20 mM Tris pH 7.5, 20 mM NaCl, 0.35 mM NaN_3_. Collected on a 600 MHx Bruker AVANCE magnet.
(TIF)

S4 Fig HSQC overlay of obscurin Ig58/59 with Ig58/59 R4444W mutant shows only local and small chemical shift changes. Human obscurin Ig5859 (black) and Ig5859 R4444W mutant (red). Conditions were at 25 ^o^C, 20 mM Tris pH 7.5, 20 mM NaCl, 0.35 mM NaN_3_.
(TIF)

S5 Fig Chemical shift changes to obscurin Ig58/Ig59 due to the p.Arg4444Trp mutation. HSQC chemical shift changes between WT Ig58/Ig59 and the p.Arg4444Trp mutant mapped on to a representative model of the dual domain Ig58/Ig59 (A) or by residue number (B). All significant changes (> 2x average chemical shift change; 20 Hz) are colored red.
(TIF)

S6 Fig Obscurin Ig58 and Ig59 are independent of each other in solution. HSQC chemical shift changes comparing individual human obscurin Ig58 and Ig59 domains and a tandem Ig58/59 construct mapped onto a Ig58/Ig59 model (A) or by residue number (B). Gray denotes residues with no data in the dual domain construct; green denotes residues with <2x standard deviation in chemical shift (under 24 Hz on a 600 HMz magnet); yellow denotes residues with 2-3x standard deviation in chemical shift (24–36 Hz); red denotes residues >3x standard deviation in chemical shift. Note that the Ig58/Ig59 model used here is for illustration purposes only, and does not represent a high-resolution structure.
(TIF)

S7 Fig Ig58, Ig59, and Ig58/59 spectra overay significantly. HSQC overlay of human obscurin Ig59 (black), Ig58 (green), and Ig5859 (red), showing that the dual domain HSQC spectrum overlays well with the spectra from both individual Ig-like obscurin domains. Conditions for each HSQC are identical, and are as follows: 25 ^o^C, 20 mM Tris pH 7.5, 20 mM NaCl, 0.35 mM NaN_3_.
(TIF)

S8 Fig Labeled HSQC of the dual Ig58/59 obscurin construct. Residues are numbered based on full length human obscurin A (accession ID: CAC44768.1)
(TIF)

S9 Fig NOE data suggest that Ig58 and Ig59 do not significantly interact. Top panel: a model of four residues in Ig58 and Ig59 that are likely to be at or near the domain-domain interface. Bottom panels: representative ^15^N-edited 3D NOESY planes (aliphatic region) of these four residues. Spectra for Ig58 and Ig59 are in blue and spectra of the dual domain Ig5859 is in orange. For clarity, the Ig5859 NOESY spectra are slightly shifted to the left of the Ig58 or Ig59 spectra. Note that the strength and size of the NOE peaks are consistent between the spectra, and no additional peaks are visible in the Ig5859 spectra.
(TIF)

S10 Fig Titin ZIg9/ZIg10 interacts with specific residues of the obscurin Ig58/Ig59 region. HSQC overlay of ^15^N-labeled obscurin Ig5859 (black) and Ig5859 with 0.5X unlabeled titin ZIg9-10 (red). Due to the large size of this complex, further titration points resulted in uninterpretable spectra. Conditions for these titrations are at 37 ^o^C, 20 mM Tris pH 7.5, 20 mM NaCl, 0.35 mM NaN_3_ for both proteins.
(TIF)

S11 Fig SPR traces of WT and mutant obscurin Ig58/Ig59 binding to titin ZIg9/ZIg10, with affinities. Sample buffers contain 50 mM NaCl, and 20 mM Tris, pH 7.5. Samples were run at 22°C. Each trace was buffer subtracted. An unrelated 100 μM BSA showed no binding to titin ZIg9/ZIg10. Insets- example fits to the data.
(TIF)

S1 Table Genes responsible for distal muscular dystrophies. (DOC)

S2 Table Human Ig59 data collection statistics. (DOC)

S3 Table NMR-derived statistics of 20 NMR structures of human Ig59. (DOC)
