# Supplementary material for: A novel FLNC frameshift and an OBSCN variant in a family with distal muscular dystrophy
Source: PLoS One. 2017 Oct 26;12(10):e0186642. doi: 10.1371/journal.pone.0186642 (PMC5657976; doi:10.1371/journal.pone.0186642)
Supplement: S1 Text — (DOC) [file pone.0186642.s001.doc]

**S1 Supporting information**

**NMR collection details**

A 2D HSQC was collected, as well as standard triple resonance experiments including HNCACB, CBCA(CO)NH, HNCO, HN(CA)CO, C(CO)NH, H(CCCO)NH, ^15^N-edited TOCSY, ^15^N-edited NOESY, ^13^C-edited NOESY, and pseudo-3D IPAP experiment for H-N residual dipolar couplings, as previously described (Rudloff et al., 2015). Both NOESY experiments used 110 ms mixing time. Most experiments were collected with 128, 64 and 1024 points in the T1, T2, and T3 dimensions, respectively. NMR data were processed with NMRPipe (Delaglio et al., 1995), extended in the indirect dimension via linear prediction, and the resulting spectra were analyzed via Sparky (Lee et al., 2015).

Standard Bruker IPAP experiments using 256 pts for each T1 dimension were used to collect RDC data in isotropic and axially-compressed 5.5% acrylamide gel samples, as previously described (Wright et al., 2009). The program PALES was used for RDC alignment tensor fitting with a calculated Aa and Ar component of 0.00163 and 0.000901, respectively (Zweckstetter, 2008). For all experiments, the ^1^H chemical shifts were referenced to external DSS, the ^13^C shifts were referenced indirectly to DSS using the frequency ratio ^13^C/^1^H = 0.251449527 and ^15^N shifts were referenced indirectly to liquid ammonia using ^15^N/^1^H = 0.101329118.

**NMR structural calculation details**

An ensemble of structures without dihedral restraints had a backbone RMSD of 0.85 Å when compared to structures with dihedral constraints (Shen et al., 2009). We attempted to further verify the structure by performing a H-D exchange experiment, however this Ig domain remains unfolded after lyophilization. Therefore, hydrogen bond constraints were not tested directly but instead were added into the structure only after the secondary structure was completely determined. Structures calculated without hydrogen bonds had an RMSD of 0.59 Å when compared to those calculated with hydrogen bonds, indicating that inclusion of these bonds did not drastically influence the overall structure. Hydrogen bond constraints of rHN-O = 1.5 Å to 2.8 Å and rN-O = 2.4 Å to 3.5 Å were included in the final stage of structure calculations, and were based off regions that were clearly in well-defined secondary structural motifs. Pseudopotentials for secondary ^13^Cα and ^13^Cß chemical shifts and a conformational database potential were included in the final simulated annealing structural calculations using the computer program XPLOR-NIH (Schwieters et al., 2003; Schwieters et al., 2006). Structures run with and without these pseudopotentials show an RMSD of 0.58 Å. The internuclear dipolar coupling (in Hz) were determined from the difference in J splitting between isotropic and radially compressed polyacrylamide, and were incorporated into the final structure calculation as previously described using an energy constant of 0.50 (Wright et al., 2005; Wright et al., 2008). A comparison of structures run with and without RDC measurements show an RMSD of 0.67 Å. Q-factors were calculated by randomly removing ≈ 10% of the N-HN RDC data, and then comparing these values to those back-calculated from the structure.

**REFERENCES**

Delaglio F, Grzesiek S, Vuister GW, Zhu G, Pfeifer J, Bax A. (1995). NMRPipe: A multidimensional spectral processing system based on UNIX pipes. J Biomol NMR. 6:277–93.

Lee W, Tonelli M, Markley JL. (2015). NMRFAM-SPARKY: enhanced software for biomolecular NMR spectroscopy. Bioinformatics. 15;31(8):1325-1327. doi: 10.1093/bioinformatics/btu830.

Rudloff MW, Woosley AN, Wright NT. 2015. Biophysical characterization of naturally occurring titin M10 mutations. Protein Sci. 24:946-955. doi: 10.1002/pro.2670.

Schwieters CD, Kuszewski JJ, Tjandra N, Clore GM. (2003). The Xplor-NIH NMR molecular structure determination package. J Magn Reson 160:65–73.

Schwieters CD, Kuszewski JJ, Marius Clore G. (2006). Using Xplor-NIH for NMR molecular structure determination. Prog. Nucl. Magn. Reson. Spectrosc. 48:47–62.

Shen Y, Delaglio F, Cornilescu G, Bax A. (2009). TALOS+: A hybrid method for predicting protein backbone torsion angles from NMR chemical shifts. J Biomol NMR 44:213–23.

Wright NT, Varney KM, Ellis KC, Markowitz J, Gitti RK, Zimmer DB, Weber DJ. (2005). The three-dimensional solution structure of Ca2+-bound S100A1 as determined by NMR spectroscopy. J Mol Biol 353:410–26.

Wright NT, Prosser BL, Varney KM, Zimmer DB, Schneider MF, Weber DJ. (2008). S100A1 and calmodulin compete for the same binding site on ryanodine receptor. J Biol Chem 283:26676–83.

Wright NT, Cannon BR, Wilder PT, Morgan MT, Varney KM, Zimmer DB, Weber DJ. (2009). Solution Structure of S100A1 Bound to the CapZ Peptide (TRTK12). J Mol Biol 386:1265–1277.

Zweckstetter M. (2008). NMR: prediction of molecular alignment from structure using the PALES software. Nat Protoc 3:679–90.
