# Supplementary figures and images for: A novel FLNC frameshift and an OBSCN variant in a family with distal muscular dystrophy
Source: PLoS One. 2017 Oct 26;12(10):e0186642. doi: 10.1371/journal.pone.0186642 (PMC5657976; doi:10.1371/journal.pone.0186642)

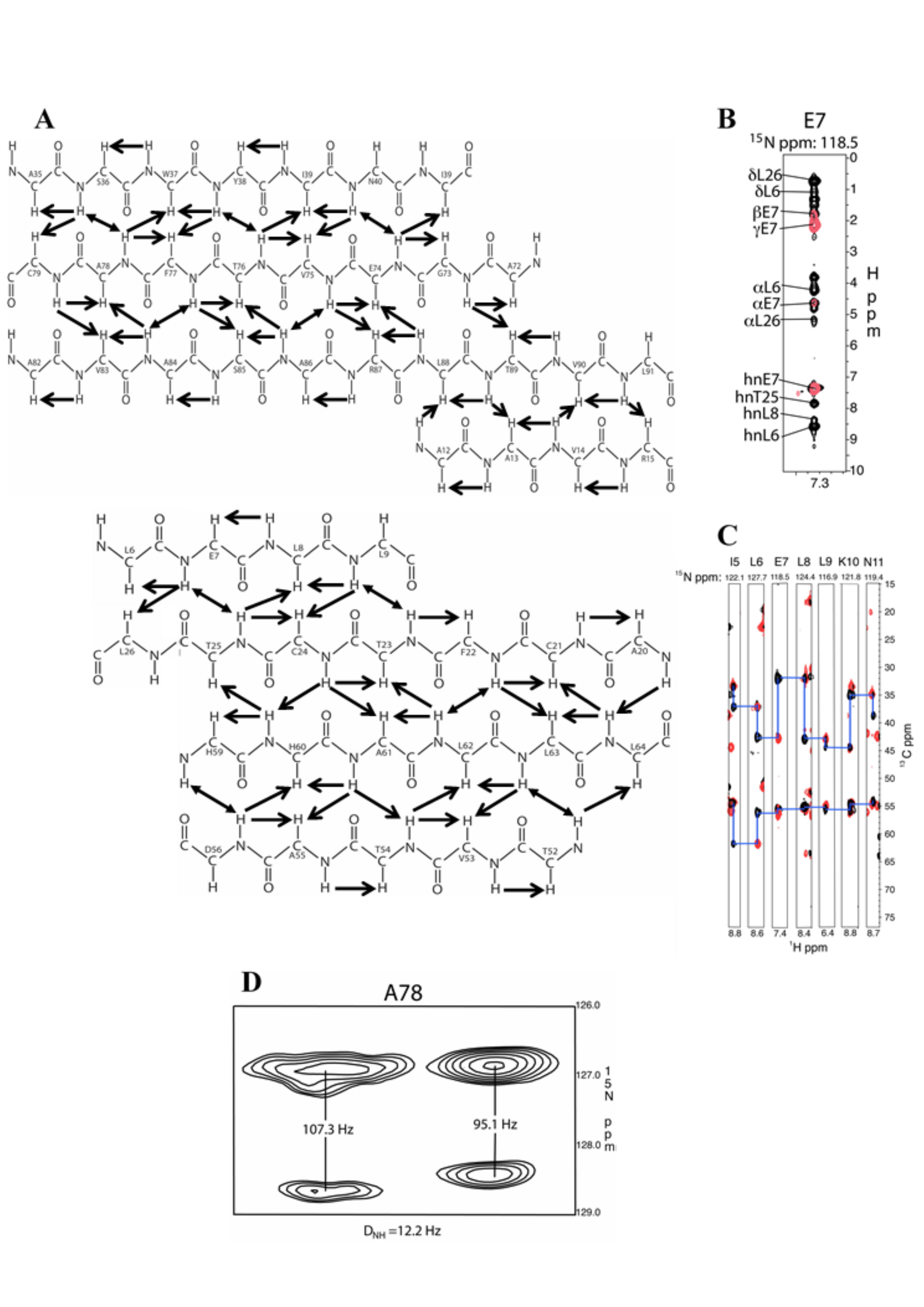

Supplement: S1 Fig — A) Observed beta sheet interactions of Ig59 as seen in the 15N-edited NOESY. B) Example of NOESY data (in black) overlaid with TOCSY data (in red), showing self-peaks and cross-strand NOEs. C) CBCA(CO)NH (in red) and HNCACB (in black) experiments, showing an example of NMR backbone assignments. D) Example of residual dipolar coupling data showing isotropic (right) and anisotropic (left) examples of the H-N bond from A78. (TIF) [file pone.0186642.s002.tif]

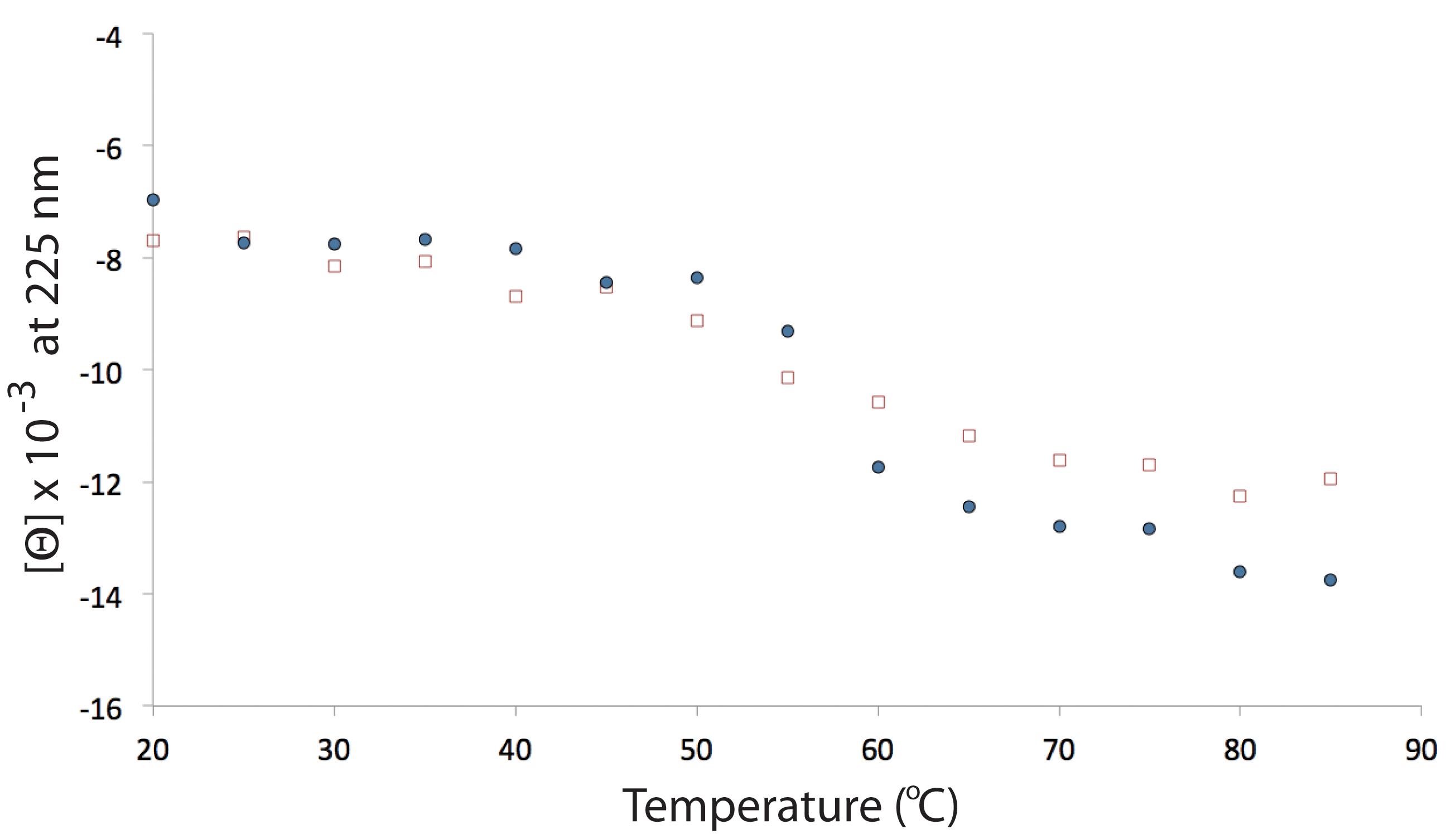

Supplement: S2 Fig — Circular Dichroism traces from 250–200 nm display a typical beta sheet-like trace, with a minimum at 217 nm. Upon heating, this peak widens (Rudloff et al., 2015). Thus, one read-out for Ig domain unfolding is CD signal at the shoulders of this 217 nm peak; as the peak widens with unfolding, this signal becomes more negative. Here, we measure the CD signal at 225 nm, using 10μM protein, 50 mM NaCl, 20 mM Tris pH 7.5 for WT (blue circle) and R444W (red square) in a 1 mm cuvette. The TM, as calculated by taking the first derivative of these data, show WT to unfolds at 58°C and the Arg4444Trp mutant to unfolds at 55°C. (TIF) [file pone.0186642.s003.tif]

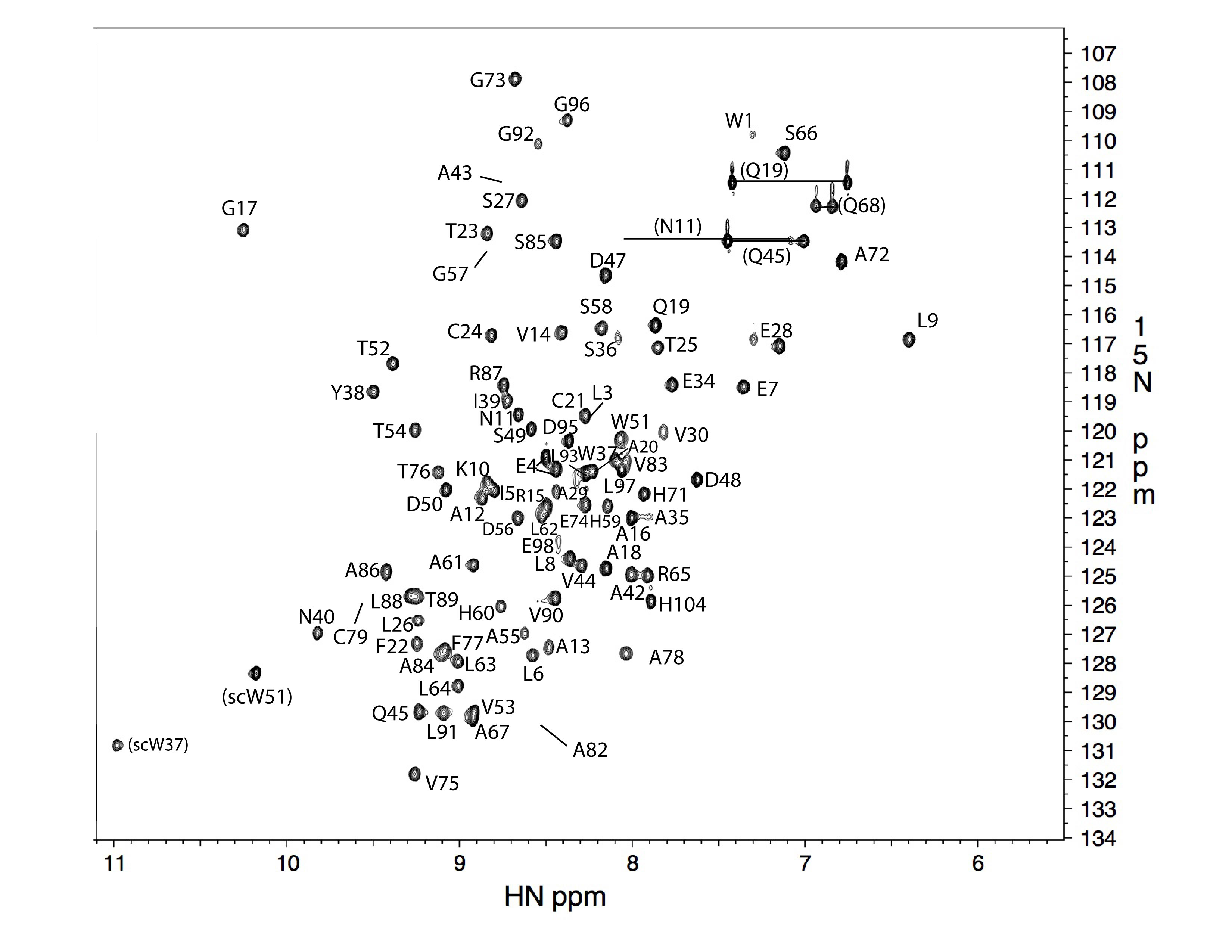

Supplement: S3 Fig — Conditions are at 25 oC, 20 mM Tris pH 7.5, 20 mM NaCl, 0.35 mM NaN3. Collected on a 600 MHx Bruker AVANCE magnet. (TIF) [file pone.0186642.s004.tif]

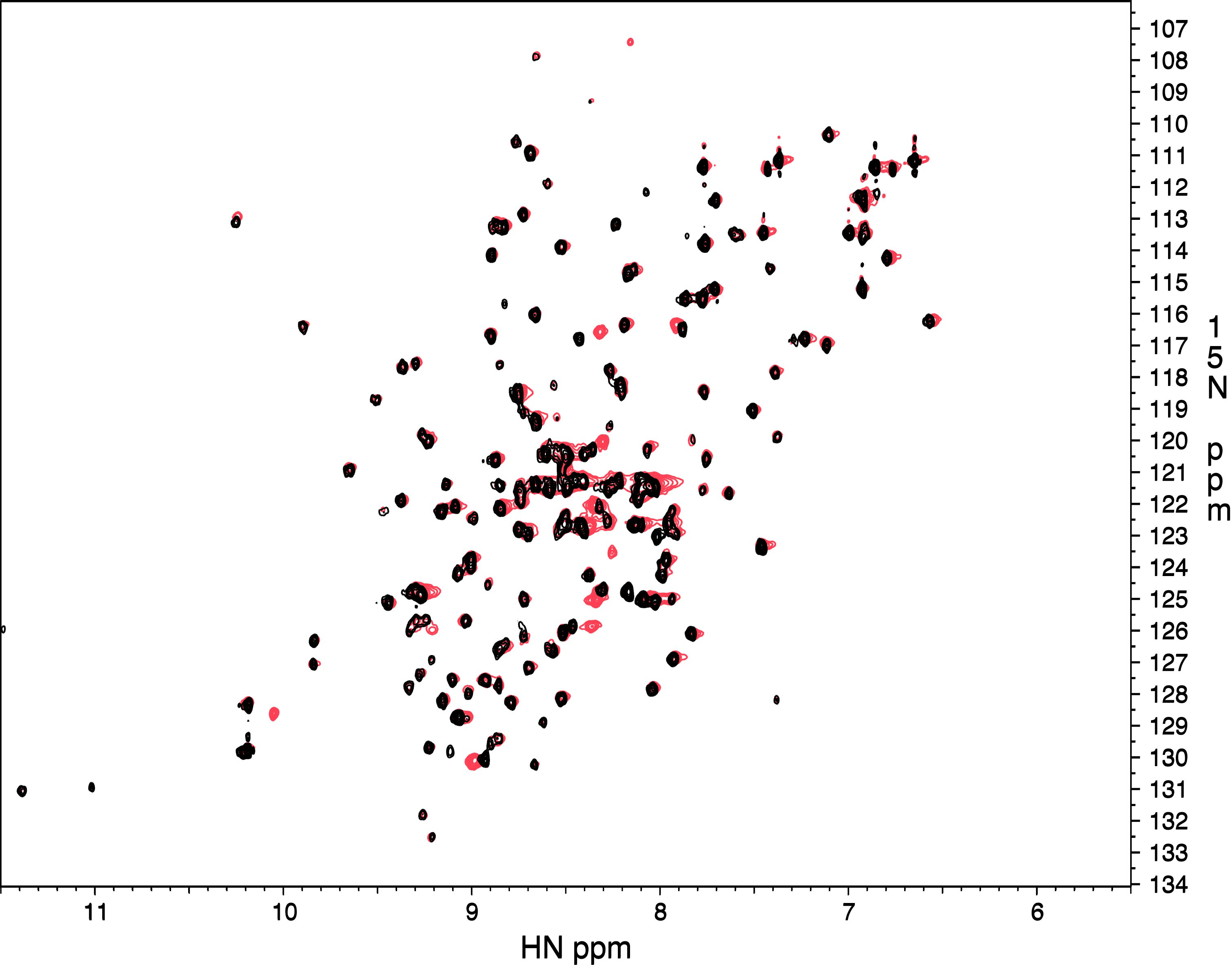

Supplement: S4 Fig — Human obscurin Ig5859 (black) and Ig5859 R4444W mutant (red). Conditions were at 25 oC, 20 mM Tris pH 7.5, 20 mM NaCl, 0.35 mM NaN3. (TIF) [file pone.0186642.s005.tif]

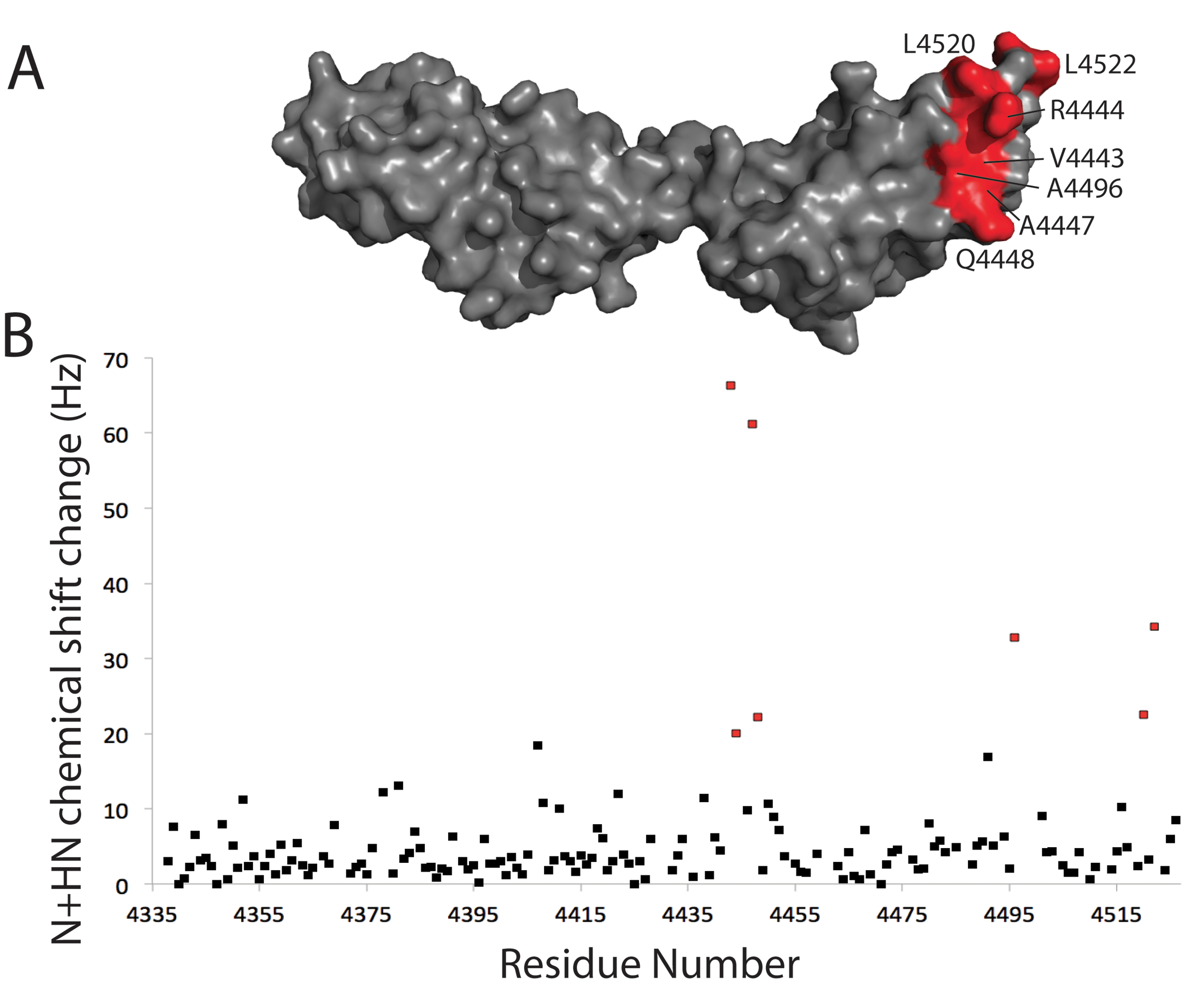

Supplement: S5 Fig — HSQC chemical shift changes between WT Ig58/Ig59 and the p.Arg4444Trp mutant mapped on to a representative model of the dual domain Ig58/Ig59 (A) or by residue number (B). All significant changes (> 2x average chemical shift change; 20 Hz) are colored red. (TIF) [file pone.0186642.s006.tif]

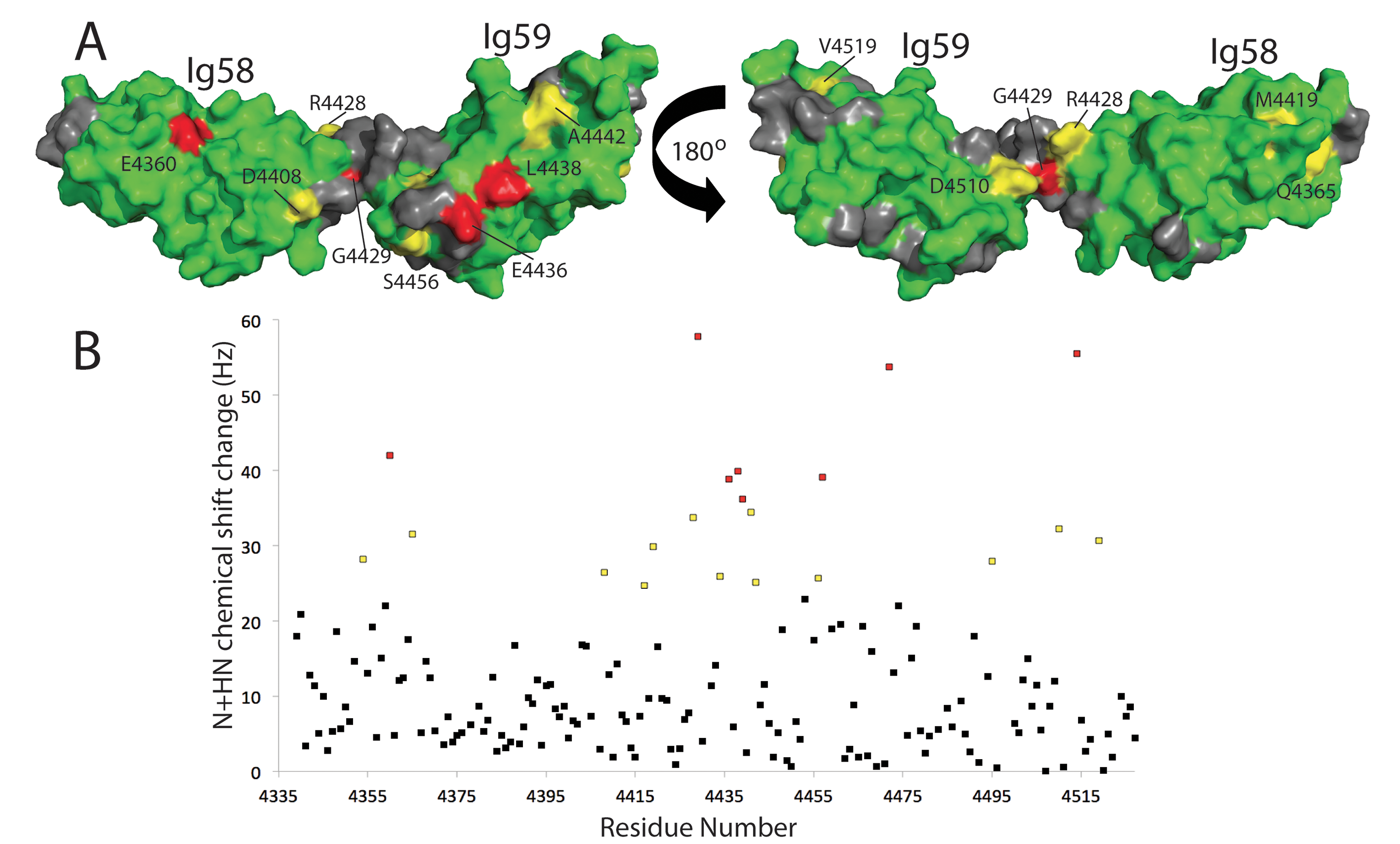

Supplement: S6 Fig — HSQC chemical shift changes comparing individual human obscurin Ig58 and Ig59 domains and a tandem Ig58/59 construct mapped onto a Ig58/Ig59 model (A) or by residue number (B). Gray denotes residues with no data in the dual domain construct; green denotes residues with <2x standard deviation in chemical shift (under 24 Hz on a 600 HMz magnet); yellow denotes residues with 2-3x standard deviation in chemical shift (24–36 Hz); red denotes residues >3x standard deviation in chemical shift. Note that the Ig58/Ig59 model used here is for illustration purposes only, and does not represent a high-resolution structure. (TIF) [file pone.0186642.s007.tif]

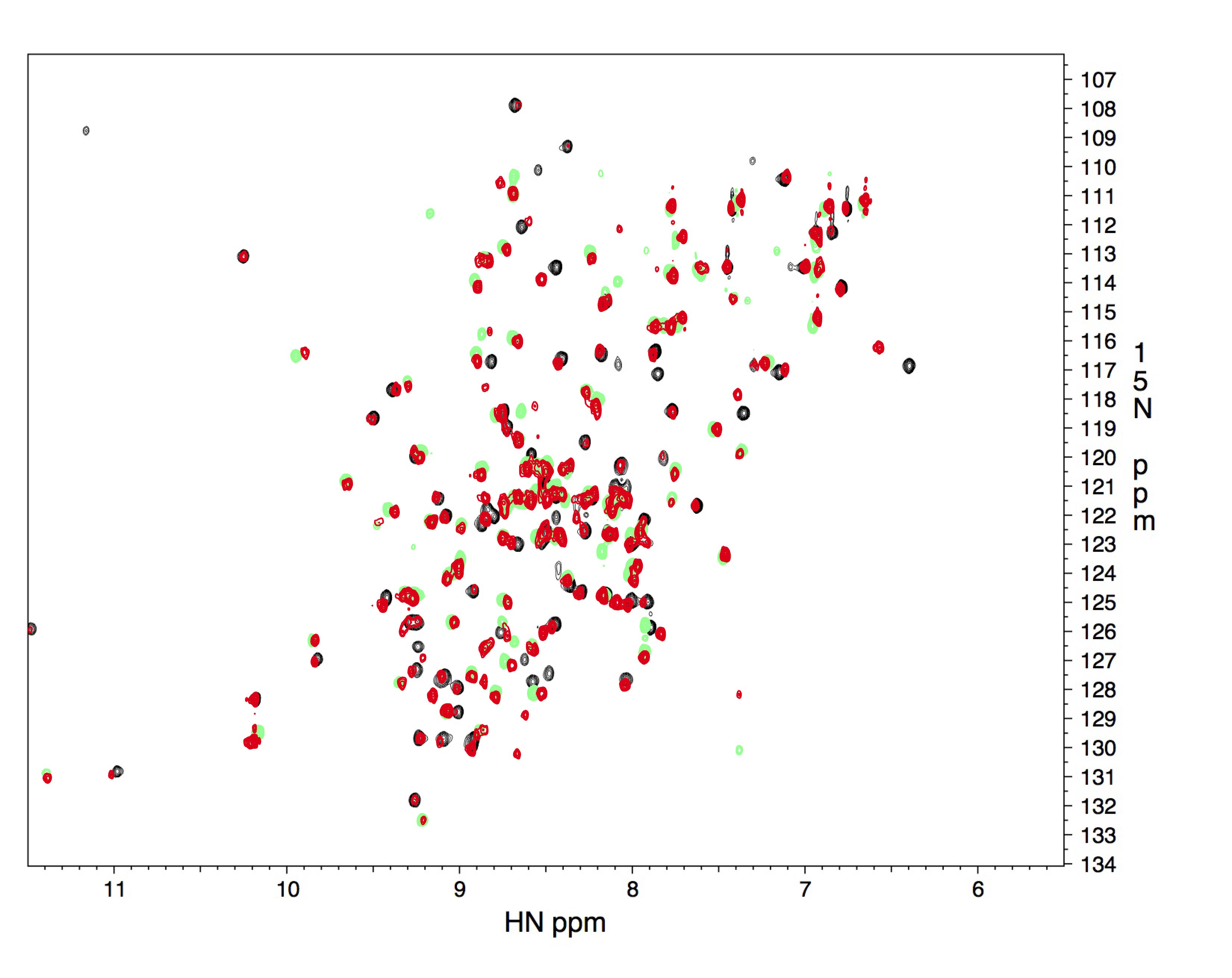

Supplement: S7 Fig — HSQC overlay of human obscurin Ig59 (black), Ig58 (green), and Ig5859 (red), showing that the dual domain HSQC spectrum overlays well with the spectra from both individual Ig-like obscurin domains. Conditions for each HSQC are identical, and are as follows: 25 oC, 20 mM Tris pH 7.5, 20 mM NaCl, 0.35 mM NaN3. (TIF) [file pone.0186642.s008.tif]

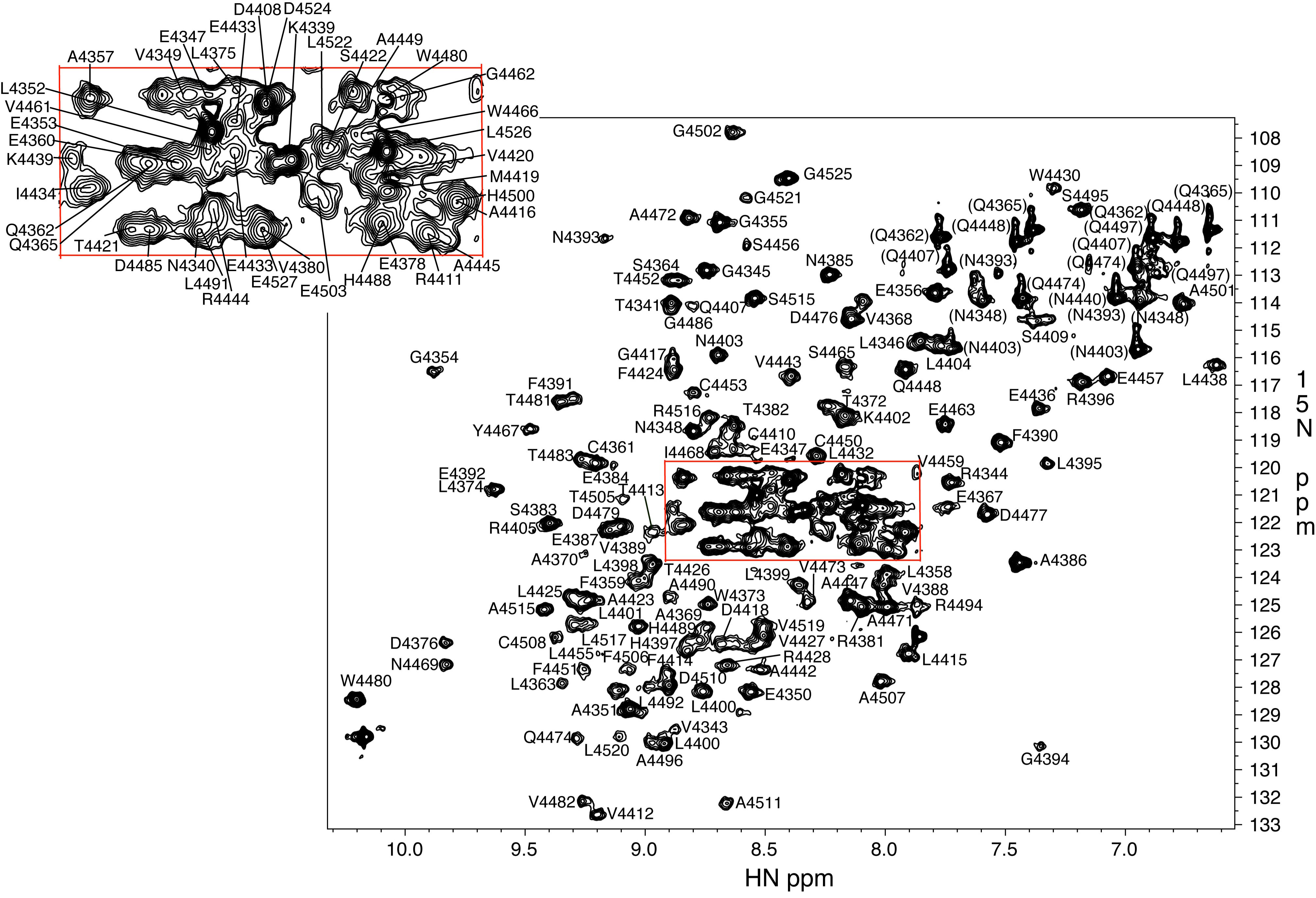

Supplement: S8 Fig — Residues are numbered based on full length human obscurin A (accession ID: CAC44768.1) (TIF) [file pone.0186642.s009.tif]

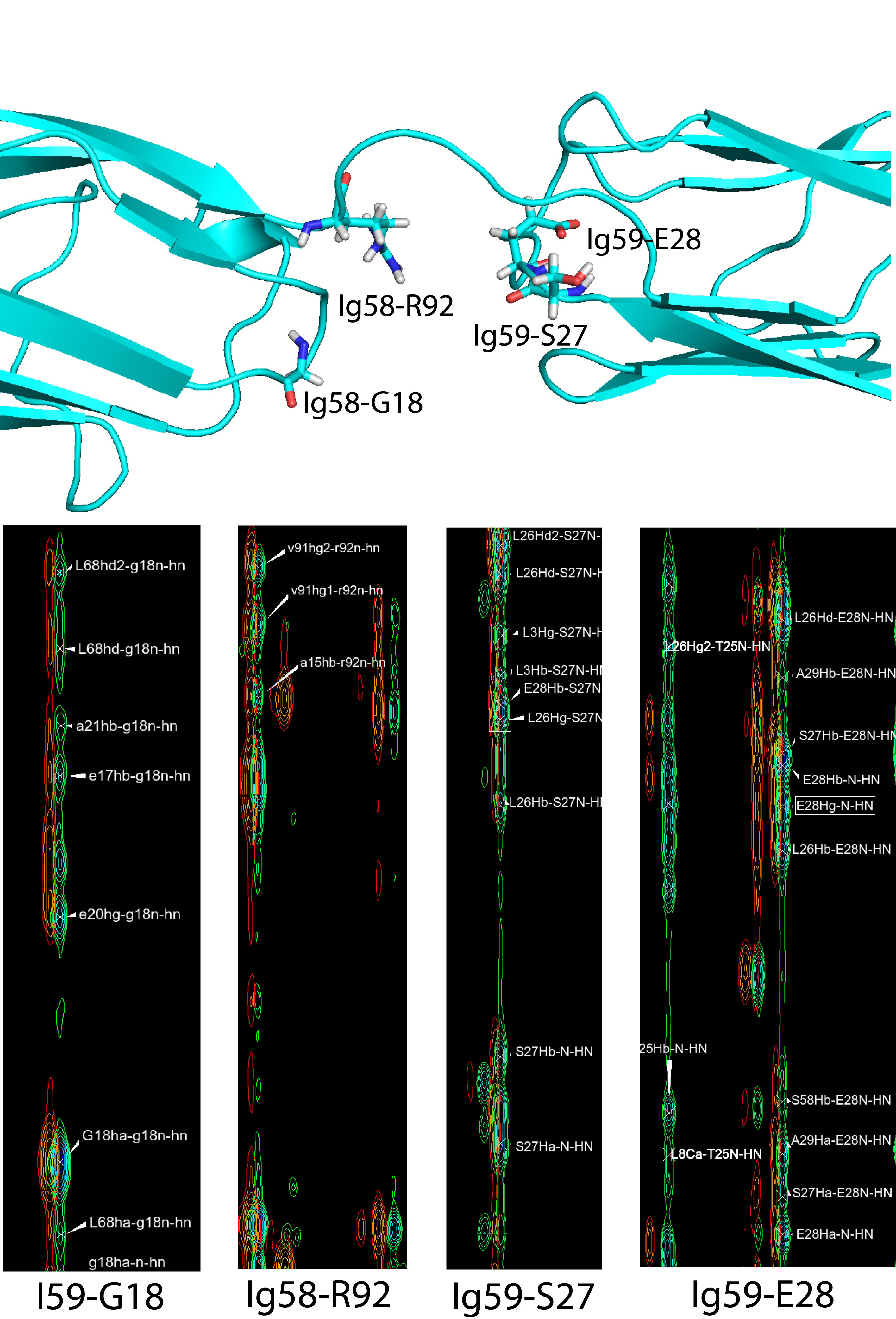

Supplement: S9 Fig — Top panel: a model of four residues in Ig58 and Ig59 that are likely to be at or near the domain-domain interface. Bottom panels: representative 15N-edited 3D NOESY planes (aliphatic region) of these four residues. Spectra for Ig58 and Ig59 are in blue and spectra of the dual domain Ig5859 is in orange. For clarity, the Ig5859 NOESY spectra are slightly shifted to the left of the Ig58 or Ig59 spectra. Note that the strength and size of the NOE peaks are consistent between the spectra, and no additional peaks are visible in the Ig5859 spectra. (TIF) [file pone.0186642.s010.tif]

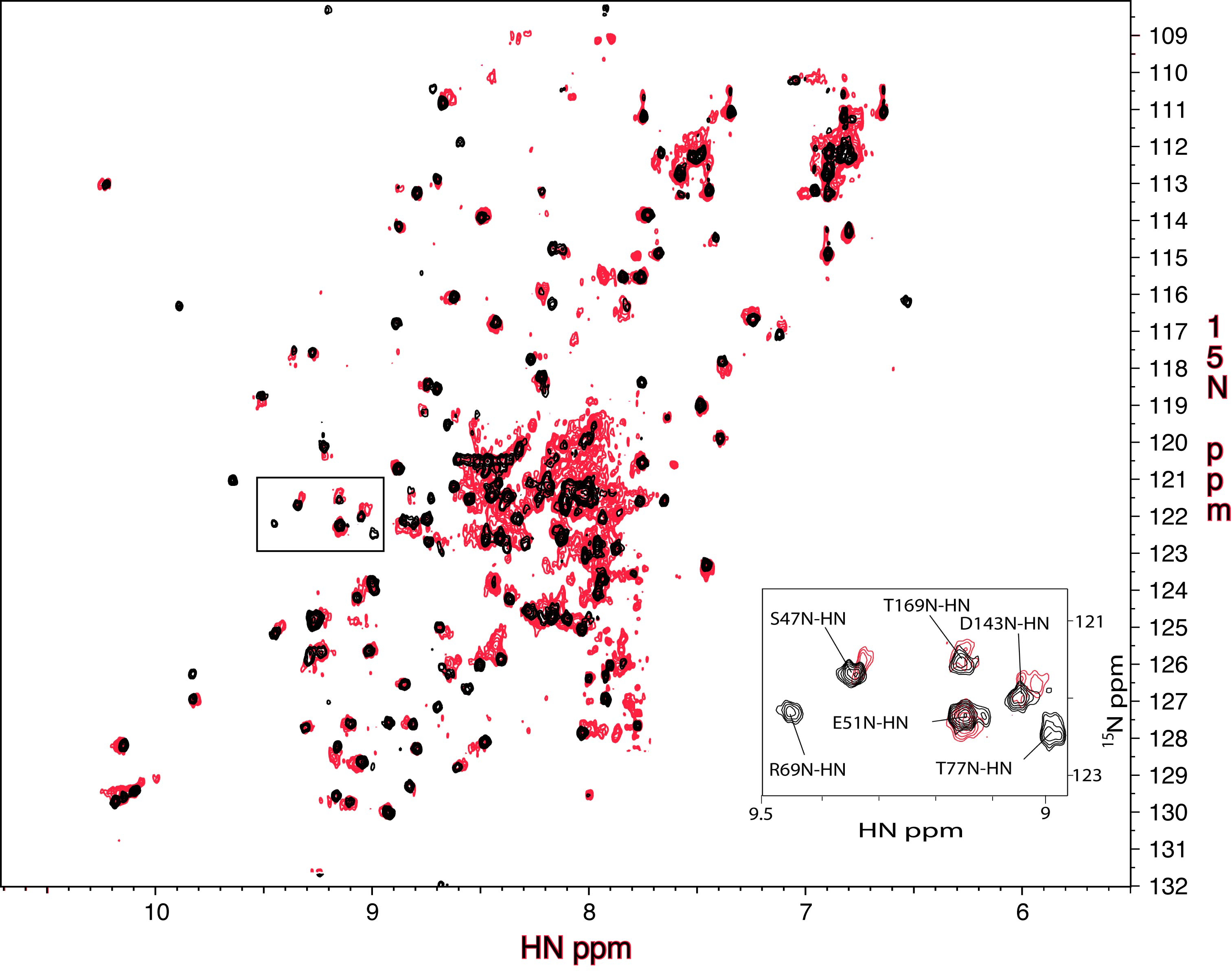

Supplement: S10 Fig — HSQC overlay of 15N-labeled obscurin Ig5859 (black) and Ig5859 with 0.5X unlabeled titin ZIg9-10 (red). Due to the large size of this complex, further titration points resulted in uninterpretable spectra. Conditions for these titrations are at 37 oC, 20 mM Tris pH 7.5, 20 mM NaCl, 0.35 mM NaN3 for both proteins. (TIF) [file pone.0186642.s011.tif]

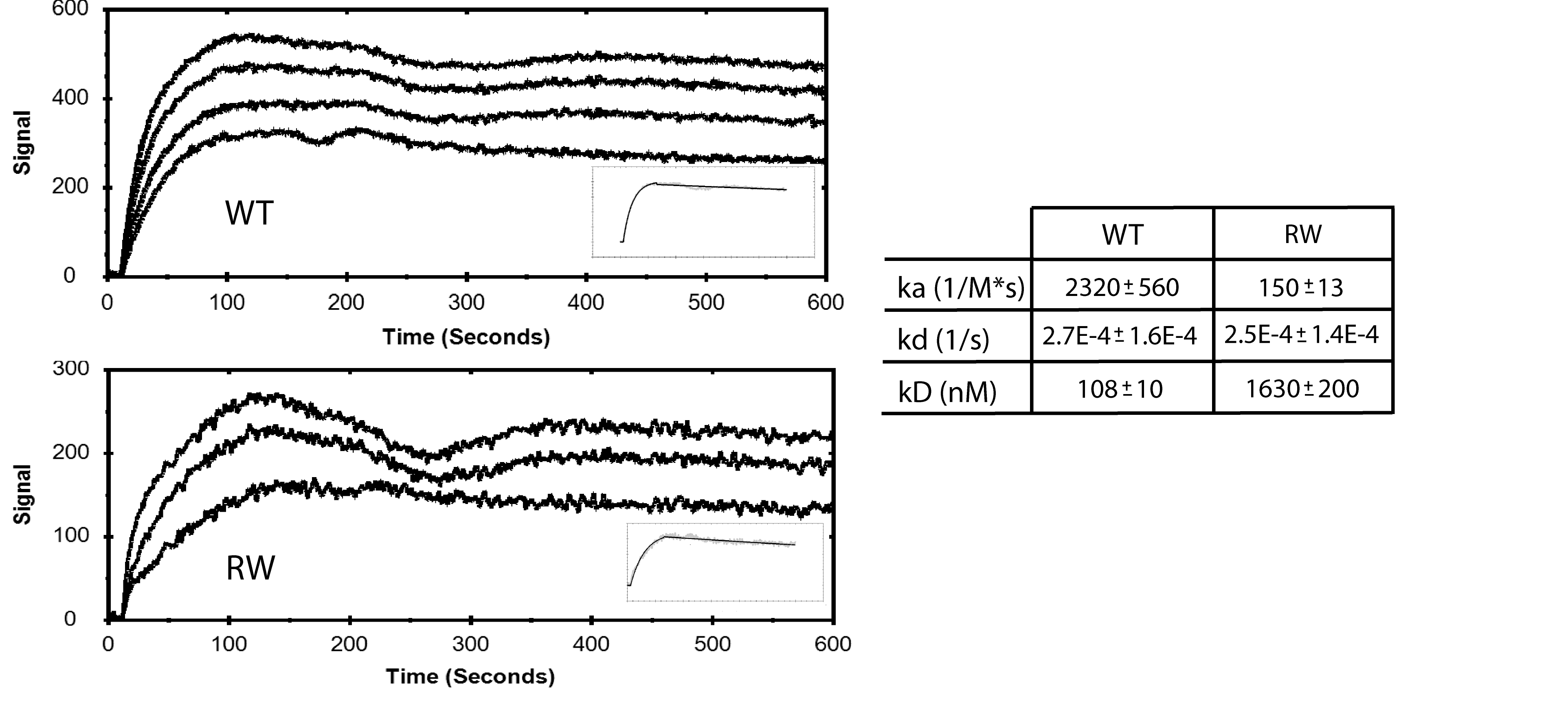

Supplement: S11 Fig — Sample buffers contain 50 mM NaCl, and 20 mM Tris, pH 7.5. Samples were run at 22°C. Each trace was buffer subtracted. An unrelated 100 μM BSA showed no binding to titin ZIg9/ZIg10. Insets- example fits to the data. (TIF) [file pone.0186642.s012.tif]
