# Supplementary material for: A novel FLNC frameshift and an OBSCN variant in a family with distal muscular dystrophy
Source: PLoS One. 2017 Oct 26;12(10):e0186642. doi: 10.1371/journal.pone.0186642 (PMC5657976; doi:10.1371/journal.pone.0186642)
Supplement: S1 Table — (DOC) [file pone.0186642.s013.doc]

**S1 Table**

- Autosomal dominant: late onset
  - TIA1 mutated Welander distal Myopathy
  - Titin mutated Tibial Muscular dystrophy
  - distal Myotilinopathy
  - ZASPopathy
  - MATR3 mutated VCPDM
  - VCP mutated distal Myopathy
  - αB-crystallin mutated distal Myopathy
  - Thenar atrophy with sarcoplasmic bodies (Edström)
  - SQSTM1 mutated distal Myopathy
- Autosomal dominant: adult onset
  - Desminopathy
  - ABD-FLNC mutated distal myopathy
  - DNAJB6 mutated distal myopathy
  - HSPB8 mutated Neuromyopathy
  - Finnish MPD3 (no gene known yet)
  - Oculopharyngeal distal myopathy (no gene known yet)
- Autosomal dominant: childhood onset
  - distal Myosinopathy MYH7
  - KLHL9 mutated distal Myopathy
- Autosomal recessive: childhood onset
  - Distal Nebulin Myopathy
  - ADSSL mutated dista1 Myopathy
  - Oculopharyngeal distal Myopathy
- Autosomal recessive: early adult onset
  - Miyoshi Dysferlinopathy
  - Distal Anoctaminopathy
  - GNE-myopathy
  - Recessive distal Titinopathy
