# Supplementary material for: A novel FLNC frameshift and an OBSCN variant in a family with distal muscular dystrophy
Source: PLoS One. 2017 Oct 26;12(10):e0186642. doi: 10.1371/journal.pone.0186642 (PMC5657976; doi:10.1371/journal.pone.0186642)
Supplement: S2 Table — (DOC) [file pone.0186642.s014.doc]

**S2 Table .** **Human Ig59 data collection statistics**

| Wavelength (Å) | 0.9792 |
| --- | --- |
| Resolution range (Å) | 30.49 - 1.18 (1.219 - 1.18) |
| Space group | P 3_1_ 2 1 |
| Unit cell | 60.98 60.98 47.56 90 90 120 |
| Total reflections | 662701 (35265) |
| Unique reflections | 33764 (3303) |
| Multiplicity | 19.5 (10.7) |
| Completeness (%) | 98.82 (92.53) |
| Mean I/sigma(I) | 22.98 (5.07) |
| Wilson B-factor | 14.8 |
| R-merge | 0.1345 (0.539) |
| R-meas | 0.1389 |
| CC1/2 | 0.988 (0.912) |
| CC* | 0.997 (0.977) |
| R-work | 0.165 (0.263) |
| R-free | 0.185 (0.297) |
| Number of non-hydrogen atoms | 775 |
| macromolecules | 678 |
| water | 97 |
| Protein residues | 90 |
| RMS(bonds) | 0.033 |
| RMS(angles) | 1.46 |
| Ramachandran favored (%) | 99 |
| Ramachandran outliers (%) | 0 |
| Clashscore | 2.23 |
| Average B-factor | 20.5 |
| macromolecules | 19.4 |
| solvent | 28.3 |
