# Supplementary material for: A novel FLNC frameshift and an OBSCN variant in a family with distal muscular dystrophy
Source: PLoS One. 2017 Oct 26;12(10):e0186642. doi: 10.1371/journal.pone.0186642 (PMC5657976; doi:10.1371/journal.pone.0186642)
Supplement: S3 Table — (DOC) [file pone.0186642.s015.doc]

**S3 Table. NMR-derived statistics of 20 NMR structures of human Ig59[[1]](#footnote-2)**

<20> best

**rmsd from distance constraints (Å)[[2]](#footnote-3)**

total (1061) 0.028 ± 0.002 0.029

intraresidue (228) 0.005 ± 0.004 0.003

sequential ( |*i – j*| = 1) (382) 0.019 ± 0.005 0.022

medium range ( 1 < |*i – j*| ≤ 1) (95) 0.046 ± 0.006 0.056

long range ( |*i – j*| = 1) (292) 0.031 ± 0.003 0.003

hydrogen bonds (64) 0.056 ± 0.006 0.050

**rmsd from exptl dihedral constraints (°)**

*Φ,Ψ* (144) 0.687 ± 0.116 0.684

**rmsd from dipolar coupling restraints (Hz)**

D_NH_  (52) 0.89 ± 0.09 0.93

**rmsd from exptl ^13^C chemical shifts**

^13^Ca (ppm) 1.59 ± 0.17 1.31

^13^Cb (ppm) 1.46 ± 0.04 1.47

**rmsd from idealized geometry**

bonds (Å) 0.004 ± 0.001 0.004

angles (**°**) 0.633 ± 0.020 0.640

impropers (**°**) 0.442 ± 0.036 0.431

**Lennard-Jones potential energy (kcal/mol)[[3]](#footnote-4)** -363 ± 10 -359

**Q-value** [[4]](#footnote-5) 0.27 ± 0.04 0.25

**% most favorable region in the Ramachandran plot [[5]](#footnote-6)** 74.1± 3.0 72.8

**rmsd of the mean structure (Å) [[6]](#footnote-7)**

all backbone atoms (3-91) 0.609 ± 0.063 0.494

all heavy atoms (3-91) 1.2128 ± 0.080 1.157

1. The 20 ensemble structures, <20>, are the results of simulated annealing calculations. The best structure is the closest to the average structure. The values shown for the <20> are the mean ± standard deviation. [↑](#footnote-ref-2)
2. None of the 20 structures has a distance violation > 0.35 Å or a dihedral angle violation of > 5°. The force constants used in the SA calculations are as follows: 1000 kcal mol ^−1^ Å^2^ for bond length, 500 kcal mol^−1^ rad^−2^ for angles and improper torsions, 4 kcal mol^−1^ Å^−4^ for the quartic van der Waals (vdw) repulsion term (hard-sphere effective vdw set to 0.8 times their values in CHARMm parameters), 50 kcal mole^−1^ Å^−2^ for experimental distance constraints, 1 kcal mol^−1^ Å^−2^ for distance symmetry constraints, 0.5 kcal mol^−1^ ppm^−2^ for the ^13^C chemical shift constraints, and 1.0 for the conformational database potential. The force constants (in kcal Hz^−2^) used for dipolar coupling restraints is 0.50. [↑](#footnote-ref-3)
3. Lennard-Jones van der Waals energies were calculated using CHARMm parameters and were not used in any stage of the structure determination [↑](#footnote-ref-4)
4. Q-values were determined by randomly removing 10% of all RDC values. To ensure accuracy, an ensemble of structures with a second randomly removed subset of RDCs was also run. The Q-value of this second set was similar to the first. [↑](#footnote-ref-5)
5. PROCHECK was utilized to generate the Ramachandran plot [↑](#footnote-ref-6)
6. Backbone calculations include C^α^, N, and C′ atoms. Only residues 3–91 are included since no long-range NOE correlations were observed for residues 1–2 and 92–104. [↑](#footnote-ref-7)
